# Genetic structure and evolutionary diversity of mating-type (*MAT*) loci in *Hypsizygus marmoreus*

**DOI:** 10.1186/s43008-021-00086-8

**Published:** 2021-12-20

**Authors:** Gang Wang, Yuanyuan Wang, Lianfu Chen, Hongbo Wang, Lin Guo, Xuan Zhou, Meijie Dou, Baiyu Wang, Jingxian Lin, Lei Liu, Zhengchao Wang, Youjin Deng, Jisen Zhang

**Affiliations:** 1 Jiangsu Key Laboratory for Bioresources of Saline Soils, Jiangsu Provincial Key Laboratory of Coastal Wetland Bioresources and Environmental Protection, Jiangsu Synthetic Innovation Center for Coastal Bio-Agriculture, Yancheng Teachers University, Yancheng, 224002 China; 2 Center for Genomics and Biotechnology, Haixia Institute of Science and Technology, Fujian Provincial Key Laboratory of Haixia Applied Plant Systems Biology, College of Life Sciences, Fujian Agriculture and Forestry University, Fuzhou, 350002 China; 3 College of Plant Sciences and Technology, Huazhong Agricultural University, Wuhan, 430000 China; 4 College of Crop Science, Fujian Agriculture and Forestry University, Fuzhou, 350002 China; 5 Provincial Key Laboratory for Developmental Biology and Neurosciences, College of Life Sciences, Fujian Normal University, Fuzhou, 350002 China

**Keywords:** Basidiomycota, *Hypsizygus marmoreus*, Sexual reproduction, Mating-type, Tetrapolar

## Abstract

The mating compatibility in fungi is generally governed by genes located within a single or two unlinked mating type (*MAT*) loci. *Hypsizygus marmoreus* is an edible mushroom in the order Agaricales with a tetrapolar system, which contains two unlinked *MAT* loci-homeodomain (*HD*) transcription factor genes and pheromone/pheromone receptor genes (*P*/*R*). In this study, we analyzed the genetic structure and diversity of *MAT* loci in tetrapolar system of *H. marmoreus* through sequencing of 54 heterokaryon and 8 homokaryon strains. Although within the *HD* loci, the gene order was conserved, the gene contents were variable, and the *HD* loci haplotypes were further classified into four types. By analyzing the structure, phylogeny, and the *HD* transmissibility based on the progeny of these four *HD* mating-type loci types, we found that they were heritable and tightly linked at the *HD* loci. The *P/R* loci genes were found to comprise three pheromone receptors, three pheromones, and two pheromone receptor-like genes. Intra- and inter-specific phylogenetic analyses of pheromone receptors revealed that the *STE3* genes were divided into three groups, and we thus theorize that they diverged before speciation. Comparative analysis of the *MAT* regions among 73 Basidiomycete species indicated that the diversity of *HD* and *P/R* loci in Agaricales and Boletales may contribute to mating compatibility. The number of *HD* genes were not correlated with the tetrapolar or bipolar systems. In *H. marmoreus*, the expression levels of these genes at *HD* and *P/R* loci of compatible strains were found higher than in those of homonuclear/homokaryotic strains, indicating that these mating genes acted as switches for mating processes. Further collinear analysis of *HD* loci in interspecific species found that *HD* loci contains conserved recombination hotspots showing major rearrangements in *Coprinopsis cinerea* and *Schizophyllum commune,* suggesting different mechanisms for evolution of physically linked *MAT* loci in these groups. It seems likely that gene rearrangements are common in Agaricales fungi around *HD* loci. Together, our study provides insights into the genomic basis of mating compatibility in *H. marmoreus*.

**Supplementary Information:**

The online version contains supplementary material available at 10.1186/s43008-021-00086-8.

## INTRODUCTION

Sexual reproduction plays a pivotal role in the biology of many eukaryotes (Dacks and Roger 1999). In the fungal kingdom, sexual reproduction occurs in different forms (Fraser et al. 2007). The molecular mechanisms involved in the sexual reproduction in Basidiomycete species are known across several species due to the availability of whole genome information. Sexual reproduction in Basidiomycetes starts with hyphal fusion and is regulated by mating-type genes (Raudaskoski 2015). These mating-type genes play a vital role in Basidiomycetes by regulating the establishment of heterokaryotic mycelia and subsequent formation of fruiting bodies. In the phylum Basidiomycota, sexual reproduction is often dictated by two sets of specific independent mating-type genes, which control different stages of sexual cycle (Maia et al. 2015). In the bipolar system, the pheromone receptor (*P/R*) and homeodomain (*HD*) genes are linked at a single *MAT* locus, resulting in the generation of only two mating types following meiosis. Alternatively, the tetrapolar system comprises two unlinked mating-type loci (Xiong et al. 2014); the *HD* loci, encoding two homeodomain-type transcription factors controlling post-mating growth, and the *P/R* loci, usually containing tightly linked pheromone and pheromone receptor genes that are involved in premating recognition (Xiong et al. 2014; Sun et al. 2019). These genes encode pre-mating lipopeptide pheromones and their cognate receptors (*P/R*), which mediate recognition of mating partners and cell fusion. Similarly, homeodomain transcription factors (*HD1* and *HD2*) form heterodimers that regulate post-mating behavior (Nieuwenhuis et al. 2013). Recombination suppression ensures full linkage of the mating-type genes within each of these two loci, required for the correct mating-type determination, and typically does not extend beyond the mating-type genes (Sara et al. 2017). It was observed that the mating types of Basidiomycete species are highly diverse and contain a large number of specific types. For a basidiomycetes species, greater the number of mating partners, the greater the chance of a mononuclear mycelium encountering another amiable mycelium, and the higher the probability of successful mating and hybridization, such as in *Lentinula edodes*, *Coprinopsis cinerea and Schizophyllum commune* (Maia et al. 2015). The number of mating types can be greatly influenced by the recombination and suppression around mating type loci, and previous studies had showed that linkage of loci involved in gamete recognition and post syngamy compatibility is responsible for derivation of a bipolar mating-type segregation from a tetrapolar system that has two independently segregating traits (Petit et al. 2012).

The molecular structures of mating-type genes were revealed adjacently in *C. cinerea* and *S. commune*, with subsequent description of *A* and *B* complex genes (Raudaskoski 2015). The structure of *HD* loci is well characterized in *C. cinerea*, in which *HD* loci contains at least three pairs of genes, including homeodomain protein families (*HD1* and *HD2*). Several common features are shared by HD1 and HD2 proteins in the composition of functional domains, including conserved dimerization domains, nuclear localization signals, and DNA binding HD domains, despite their differences in size and sequence (Spit et al. 1998). In particular, the N-termini of HD proteins are highly variable. Polar and hydrophobic interactions between N-terminal regions enable the heterodimerization of HD proteins and preventing homodimerization (Banham et al. 1995; Kämper et al. 1995; Ha et al. 2018). Unlike *L. edodes*, in most studied species, the *HD* loci are flanked by a gene encoding for a mitochondrial intermediate peptidase (*Mip*) (Bao et al. 2013) that is closely linked to the *HD* loci (Au et al. 2014). The beta-flanking gene (*Bfg*) is another conserved gene that flanks the opposite side of the *HD* loci in most species, except for *S. commune* (Ohm et al. 2010; Rong et al. 2015)*.*

*P/R* loci have one or more subsites, each of which contains one or more *P/R* genes. The pheromone receptors were classified within the Rhodopsin-like superfamily and typically contain seven membrane-spanning regions (7-TM). They were further characterized by a short N-terminal extracellular domain and a long cytoplasmic C-terminal tail (Dohlman et al. 1991). Additionally, the pheromone receptors are encoded by G protein-coupled receptors (GPCRs), comprised of seven transmembrane domains with short extracellular and long intracellular parts, and are associated with *α*-subunit of heterotrimeric G-protein (Wendland et al. 1995; Vaillancourt et al. 1997; Riquelme et al. 2005; Raudaskoski 2015). The phosphorylation of G-proteins is triggered by the interaction of specific pheromones with extracellular pheromone receptor domains (van Peer et al. 2011). Pheromone precursors encode 40–100 amino acid long peptides, and mature through farnesylation of the C-terminal CAAX motif (C = cysteine, A = aliphatic, and X is any residue) (Wang et al. 2016). They usually contain an acidic amino acid pair as well (ER or EH in *C. cinerea*) about 10–15 amino acids from the C-terminus (van Peer et al. 2011).

*H. marmoreus* is a wood rotting fungi that grows on stems and roots of broad-leaved trees such as beeches. It is one of the most popular and widely cultivated edible mushrooms worldwide. Like many other species of Basidiomycetes such as *C. cinerea*, *S. commune*, *Pleurotus djamor*, and *Flammulina velutipes*, *H. marmoreus* also has a tetrapolar mating system with multiple alleles (Spit et al. 1998; Kües 2000; James et al. 2004b; Ohm et al. 2010; Yu et al. 2021), but the genetics of its mating-type system is still unknown. The goals of this study are to 1) identify the genetic structure and explore diversity of the mating type loci in the tetrapolar system in *H. marmoreus*, 2) understand the evolution of the mating type loci of *H. marmoreus* in fungi, and 3) provide clues for compatible mating patterns of MAT in *H. marmoreus*.

## MATERIALS AND METHODS

### *H. marmoreus* strains and growth conditions

All 54 heterokaryotic strains of *H. marmoreus* used in this study are shown in Additional file 1: Table S1. These strains were collected from different scientific research institutes, universities and enterprises. The single spore of strain HM62-W was taken from a fruiting body. Through heterokaryon cultivation, monokaryotic spores were obtained from the pileus of fruiting bodies. The basidiospores were collected from strain HM61 and HM88, respectively, and through hybridization HMZ5 and HMZ2 hybrid strains were obtained. The basidiospore HMZ5_07, HMZ5_09, HMZ5_21, and HMZ5_78 were isolated from dikaryon strain HMZ5. The basidiospore HM68_3, HM68_4 were isolated from dikaryon strain HM68. HM61_G6 and HM88_W2 were derived from HM61 and HM88 respectively. The common data from NCBI used in this study is shown in Additional file 2: Table S2. All mononuclear and dikaryotic hyphae were cultured on potato dextrose agar (PDA) medium in Petri plates and incubated at 24 °C for two weeks. For the fruiting bodies assays, each strain was cultivated on substrate medium containing sawdust, cottonseed shell, and wheat bran with 65% water content. The pure culture of *H. marmoreus* strains were collected from Fuquanxin Biotechnology Co., LTD., Wanchen Biotechnology Co., LTD., Shenlong Mushroom Industry Co., LTD., and the Center for Genomics and Biotechnology, Fujian Agriculture and Forestry University (Fuzhou, China).

### Genome sequencing and annotation

A high-quality genome of *H. marmoreus* HM62-W is available in our laboratory (JABWDO000000000.1) and used as reference genome (Wang et al. 2021). A total of 56 dikaryotic *H. marmoreus* strains and 8 single spore isolates (SSIs) were grown on substrate, harvested, washed thrice with sterile deionized water, and stored at -80 °C for further study. The freeze-dried mycelia were grounded with liquid nitrogen and total genomic DNA was extracted using the CTAB method as previously described by Manicom et al. (1987). All 64 strains were sequenced with Illumina Hiseq2500 platform for preparation of Illumina paired-end library with an insert size of ~ 450 bp, producing 150 bp short reads of up to ~ 3 Gb of raw data, which is available as Sequence Read Archive (SRA) in NCBI under accession number (PRJNA508399 and PRJNA644211). The complete raw data of genome resequencing of these 64 strains were preprocessed by Trimmomatic for quality control and cut off of low quality reads (Bolger et al. 2014), and clean data was de novo assembled using IDBA software (Peng et al. 2012). An automatic genome-wide annotation tool GETA (https://github.com/chenlianfu/geta) was used to carry out gene prediction of these 64 strains.

### The mating-type gene identification and structure prediction

We downloaded the published protein sequences of *HD* mating-type genes and pheromone receptors of *C. cinerea*, *S. commune*, *L. bicolor,* and *L. edodes* from NCBI (Additional file 2: Table S2). The protein sequences of *HD* mating-type genes were searched by BLASTP from genomes of HM62-W and other 64 *H. marmoreus* strains. *Mip* and *Bfg* are the flanking genes that determine *HD* mating-type genes and their sequence position*.* The coiled-coil dimerization motifs (CCDs) of HD proteins were identified using COILS (window size 14, http://www.ch.embnet.org/software/COILS_form.html) (Lupas et al. 1991), the nuclear localization signal (NLS) using PSORT II (http://psort.hgc.jp/form2.html) (Nakai and Kanehisa 1992), and *HDs* using InterPro (http://www.ebi.ac.uk/interpro/). Alignment of DNA sequences were performed using DNAMAN (Huang and Zhang 2004).

Basidiomycete pheromone precursors generally consist of more than 20 amino acid residues and the sequence of genes encoding pheromone precursors are very short, with high variability. We used Perl programs to perform ORF searches 5 kb to left and right positions of pheromone receptor genes in the genome. The searched ORF information was compared with the HMM database of the PF08015 gene family to obtain pheromone precursor ORF, and we further identified whether it was a pheromone gene based on specific CAAX structure of the pheromone precursor.

### Phylogenetic tree analysis of the *HD* and *P/R* loci genes

The phylogenetic tree for interspecific Basidiomycetes and intraspecific *H. marmoreus* populations were constructed for *HD* mating-type genes and pheromone receptors by using MEGA7.0 software with maximum likelihood (ML) (Kumar et al. 2016). The branch values from 1,000 nonparametric bootstrap repetitions were used for nodal support, and nodes considered were strongly supported with bootstrap-value > 70%. In addition, a phylogenetic tree was constructed based on the whole genome of six Basidiomycetes (*L. bicolor*: PRJNA550851, *L. edodes*: PRJNA453846, *P. chrysosporium*: PRJNA495103, *S. commune*: PRJNA392531, *S. lacrymans*: PRJNA412961, *C. cinerea*: PRJNA1447), and the homologous protein sequences of all seven species were aligned using the MAFFT software (Katoh and Standley 2013). Then, the results were analyzed by OrthoMCL (Li et al. 2003) with default parameters to obtain orthologous genes. Multiple sequence alignments were calculated using the MAFFT software (Katoh and Standley 2013) and combined into a long sequence for each species. Gblocks software was used with default parameters to pick the conserved block regions of alignment (Dereeper et al. 2010). Maximum likelihood topology searches were completed with RAxML8.1.24 using the model “PROTGAMMALGX” (Stamatakis 2014). The analyses were conducted with 1000 bootstrap replicates, and the tree was visualized using FigTree1.4.2 (http://tree.bio.ed.ac.uk/software/).

### Collinearity analysis of *HD* mating-type loci

The *HD* loci were compared with those of *H. marmoreus*, *L. edodes*, *S. lacrymans, S. commune, C. cinerea, L. bicolor, and P. chysosporium* using BLASTP to collect the collinear homologous gene location information. The ChromoMapper software was used for interactive visualization of chromosomes (Anand 2019).

### RNA extraction, sequencing, mapping, and annotation analysis

RNA was extracted from monokaryotic mycelium HM88-W2, HM61-G6 and dikaryotic mycelium HMZ5 using E.Z.N.A Plant RNA extraction Kit (Omega, Biotech, Norcross, GA, USA) by following the manufacturer’s instructions. RNA quality was quantified at OD A_260_/A_280 nm_ 1.9–2.1, and OD A_260_/A_230_ > 2.0 using NanoDrop spectrophotometer ND2000 (Thermo Scientific) and stored at -80 °C for further analysis. Total RNA samples were sequenced on an Illumina HiSeqTM 2500 platform (Illumina Inc., CA, USA) by Novogene Bioinformatics Technology Co., Ltd. (Beijing, China).

The raw sequencing data were filtered through Trimmomatic software to remove adapters and low-quality reads (PRJNA765720), and clean RNA-Seq reads after quality control were mapped onto the *H. marmoreus* genome using TopHat 2 (Kim et al. 2013). Transcript expression levels of individual genes were quantified based on the fragments per kilobase of exon per million fragments mapped (FPKM) values by using Trinity (Haas et al. 2013). Differentially expressed genes (DEGs) were identified through comparison of expression levels to infer their transcriptional changes. Following the annotation of expression levels, we identified genes with a fold change ≥ 2 and a false discovery rate (FDR) < 0.05 as significant DEGs. To determine the main biological functions of DEGs, all expressed genes were functionally annotated using the Kyoto Encyclopedia of Genes and Genomes (KEGG) database and Gene Ontology (GO). Pathway enrichment analysis was used to identify the significantly enriched metabolic pathways or signal transduction pathways for the DEGs.

## RESULTS

### *MAT-A* genes and their diversity in* H. marmoreus*

**Fig. 1:**
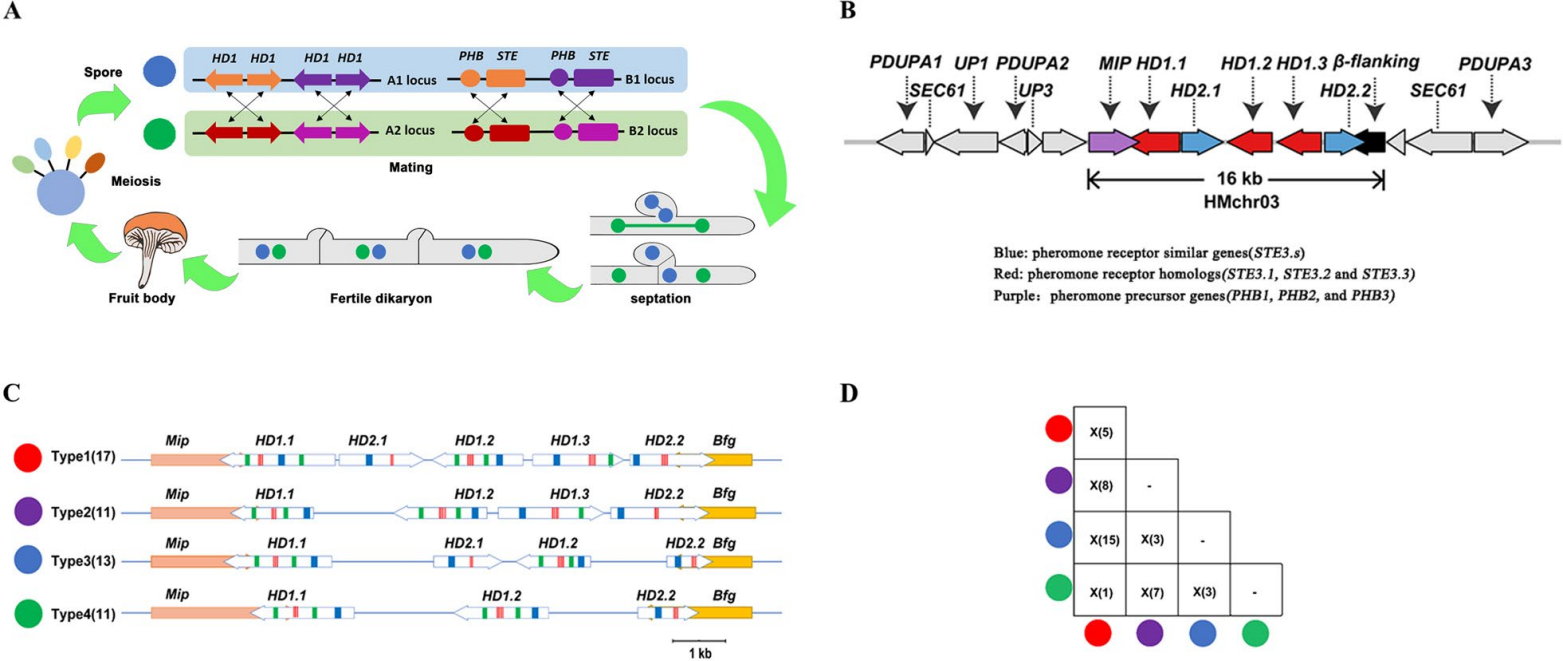
The structures and types of mating *A* loci in *H. marmoreus*. **A** The life cycle model and tetrapolar mating system of *H. marmoreus*. *HD*: Homeodomains; PHB: pheromone precursor genes; STE: pheromone receptor homologs. The direction of the arrow represents the direction of the gene. **B** The mating *HD* loci structures. The gene names shown above each arrow. **C** The type of mating *A* factor. The blue box represents Homeodomains (*HD*); the green box represents Dimerization motif (Di); the red box represents nuclear localization signals (NLS). The direction of the arrow represents the direction of the gene. **D** The number of the heterokaryon strains that contain HD mating-type haplotypes. Based on the re-sequencing of 56 dikaryotic strains, 42 of them have two HD mating-type haplotypes. Eleven strains were Type 1 & Type 2, 15 strains were Type 1 & Type 3, three strains were Type 2 & Type 3, seven strains were Type 2 & Type 4, one strain was Type 1 & Type 4, and five strains were Type 1 & Type 1. Different colors represent different HD mating type haplotypes

*H. marmoreus* has a tetrapolar mating system with multiple alleles. In the life cycle of *H. marmoreus*, the A and B mating-type genes determine compatibility in mating by regulating alternate stages in the formation of the dikaryon, an extended mycelial stage that gives rise to the fruit body (Fig. 1). Based on the reference mating-type proteins from *C. cinerea*, *S. commune,* and *L. edodes* (Ohm et al. 2010; Chen et al. 2016b), *HD* mating-type genes were searched by BLASTN and BLASTP homology from gene and protein sequence of the chromosomal-scale genome of Hm62-W. Using the mitochondrial intermediate peptidase (*Mip*), beta flanking gene (*Bfg*) as flags, three *HD1* genes (*HD1.1, HD1.2,* and *HD1.3*), and two *HD2* genes (*HD2.1* and *HD2.2*) were identified on a 16 Kbp region on chromosome 03 in *H. marmoreus*. These five *HD*s were arranged in a linear fashion and grouped into two pairs of homeodomain transcription factor genes (Fig. 1), similar to the typical *HD1*/*HD2* couplex in Basidiomycete species (Niculita-Hirzel et al. 2008; Stajich et al. 2010). The *Mip* gene was closely linked to *HD1.1* genes, whereas the *Bfg* gene was closely linked to *HD2.2* at the other end, similar to other fungi in Agaricomycetes (James et al. 2004a), excluding *S. commune* and *L. edodes* (Ohm et al. 2010).

We further assembled and annotated the genome of eight single spore isolates (SSIs) and 54 different dikaryon strains based on Illumina reads, and searched for homologous *HD* and *P/R* Mating-type genes. Consistent with the reference genome *Mip* and *Bfg* were separately located at the two ends of the *HD* loci in all strains. The alignments of *HD* loci regions showed that these strains lack overall synteny with large gaps (Additional file 6: Fig. S1). Further analysis showed that *HD* loci did not contain all 5 *HD* genes in all 54 *H. marmoreus* strains and some *HD* genes were missing for except *HD1.1* and *HD2.2*, which may contribute to the diversity of HD mating-type locus. Of the 52 complete mating sites (*Mip-HDs-Bfg*), 17 harbored all five *HD* genes (*HD1.1, HD1.2, HD1.3, HD2.1,* and *HD2.2*), 11 haplotypes harbored four *HD* genes excluding *HD2.1*, 13 haplotypes harbored four *HDs* excluding *HD2.2*, and 11 harbored 3 *HDs* without *HD2.1* and *HD1.3* were found in dikaryons (Additional file 1: Table S1) and monokaryon (Fig. 1, Additional file 4: Table S4 and Additional file 6: Fig. S2). Three *HDs*, *HD1.1*, *HD1.2,* and *HD2.2* were conserved among the *HD* of haplotypes, whereas the *HD1.1* and *HD2.2* genes were conserved likely due to the diversity of *HD* mating-type loci. Therefore, we classified the *HD* mating-type loci haplotypes into four types, Type 1: *HD1.1*, *HD2.1*, *HD1.2*, *HD1.3*, *HD2.2*; Type 2: *HD1.1*, *HD1.2*, *HD1.3*, *HD2.2*; Type 3: *HD1.1*, *HD2.1*, *HD1.2*, *HD2.2*; Type 4: *HD1.1*, *HD1.2*, *HD2.2* (Fig. 1B and Additional file 6: Fig. S2). The gene and haplotype sizes in each type of these four mating-types loci of strains *H. marmoreus* were comparable (Additional file 4: Table S4), which support the classification of these HD mating-type haplotypes.

The homeodomain identified proteins as putative transcription factors was located on N termini of HD1 and HD2 proteins. Two to four nuclear localization sequences (NLSs) were observed in *HD1.1*, *HD1.2*, and *HD1.3*. In *HD2* genes, the *HD2.1* protein contained two predicted NLSs, whereas *HD2.2* contained three to four NLSs (Fig. 1B). The locations of NLSs were consistent with HD1 and HD2 proteins across all strains. In addition, the protein sizes of these five *HD* (*HD1.1, HD1.2, HD1.3, HD2.1,* and *HD2.2*) genes were different, but each of these *HD* alleles was generally consistent across the haplotypes of the same HD mating-type.

### Mating compatibility in *H. marmoreus*

According to the above analysis, there are four types (Type 1, Type 2, Type 3, and Type 4) of *HD* mating-type loci in *H. marmoreus*. To investigate the genomic basis of mating compatibility in *H. marmoreus*, we analyzed the haplotype types of *HD* loci in dikaryotic strains based on the reference genome sequence (Hm62-W). Among all sequenced strains, 8 dikaryotic strains contained Type 1 and 2, 15 dikaryotic strains contained Type 1 and 3; 3 dikaryotic strains contained Type 2 and 3; 7 dikaryotic strains contained Type 2 and 4; 3 dikaryotic strains contained Type 3 and 4; and 5 dikaryotic strains contained only Type 1, respectively (Fig. 1C), which showed that Type 1 also contains the different *MAT-A* factor. Of note, except Type 1, none of the dikaryotic strains contained two of the same haplotypes (Type 2, 3, and 4) in *HD* loci, concurrently. These results suggested that mating compatibility was lost in the absence of both *HD2.1* and *HD1.3* in dikaryotic strains of *H. marmoreus*.

To verify the mating compatibility in *H. marmoreus*, we performed the hybridization of two spores (Hm88-W2 (Type 4) and Hm61-G6 (Type 3)) and then obtained the dikaryon HMZ5 spore. Four basidiospores of F2 generation, HMZ5_07 (A_1_B_1_, Type 4), HMZ5_78 (A_1_B_2_, Type 4), HMZ5_09 (A_2_B_1_, Type 3), and HMZ5_21 (A_2_B_2_, Type 3), were collected simultaneously, and hybridization results showed that HMZ5_07 paired with HMZ5_21 and HMZ5_09 paired with HMZ5_78. Genome sequencing and annotation revealed that the Type 4 haplotype of *HD* mating-type HM88-W2 was transferred to F1 HMZ5 and F2 HMZ5_07 and HMZ5_78, whereas the other strain HM61-G6 (Type 3) contained genes that were completely transferred to SSIs single spore isolates HMZ5_21 and HMZ5_09 by heterozygote HMZ5. These results revealed that the mating compatibility was related to the linkage of *HD* mating-type genes.

### Phylogenetic analysis of the *HD* proteins

**Fig. 2:**
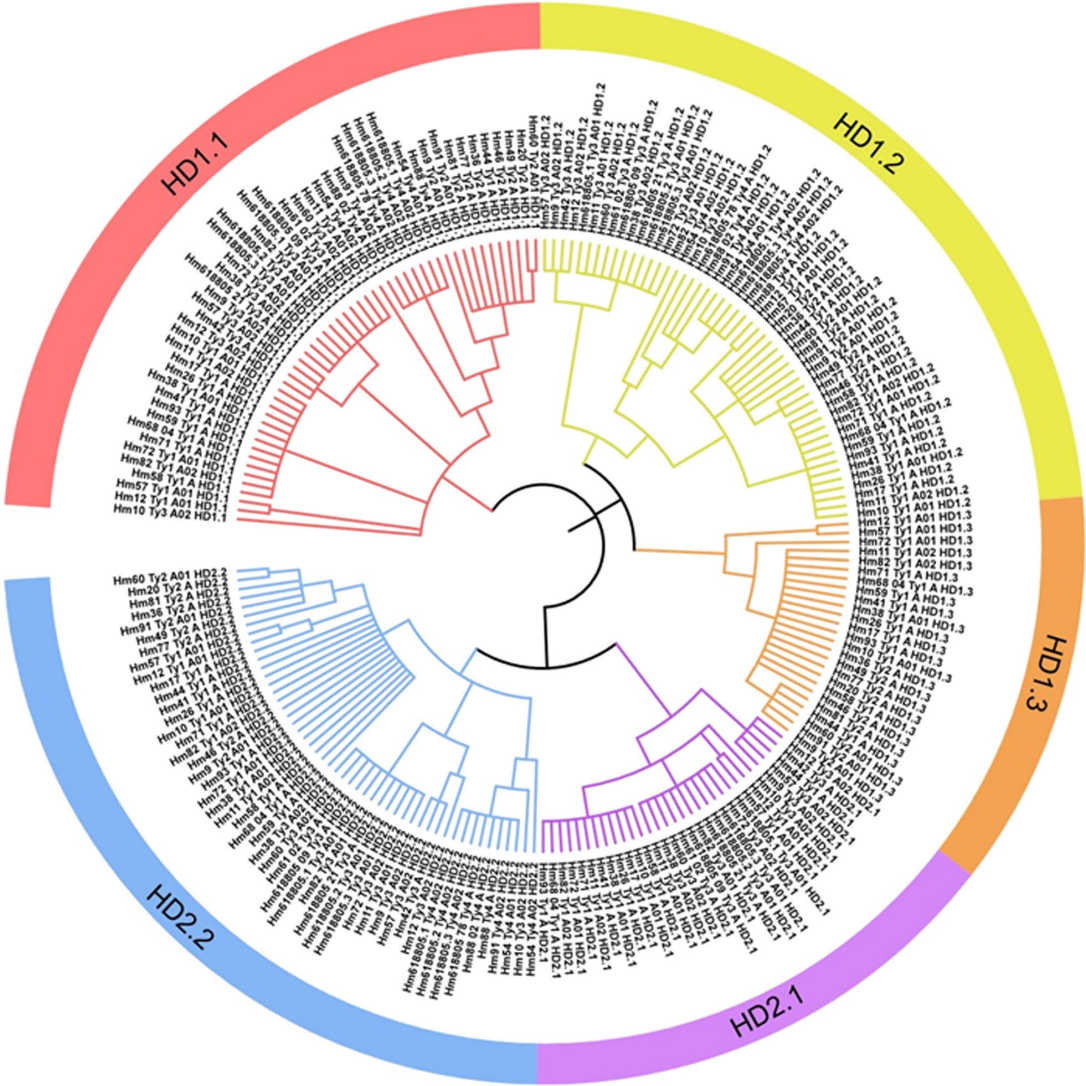
The phylogenetic trees based on 223 *HD* genes from 54 *H. marmoreus* strains. Different color blocks represent three *HD1s* and two *HD2s*

To understand the divergence of HD mating-type haplotypes in the *H. marmoreus* population, different strains of *H. marmoreus* such as 57 *HD1.1*, 56 *HD1.2*, 23 *HD1.3*, 30 *HD2.1,* and 57 *HD2.2* were used to construct phylogenetic trees according to the maximum likelihood (ML) method (Fig. 2), which demonstrated that *HD* genes were separated into two branches. The *HD1* genes (*HD1.1, HD1.2,* and *HD1.3*) were clustered in one branch and *HD2* genes (*HD2.1* and *HD2.2*) were clustered in another. These two branches were further classified into five subgroups corresponding to the five above mentioned *HD* genes (*HD1.1, HD1.2, HD1.3, HD2.1,* and *HD2.2*). Furthermore, the haplotypes of *HD* genes from each type of the four HD mating-type loci were generally phylogenetically distributed together (Additional file 6: Fig. S3–S7). The *HD1.1* and *HD1.2* genes were divided into four groups corresponding to four types of *HD* mating-type loci (Additional file 6: Fig. S3, S4). The HD1.3 proteins were classified into two groups as *HD1.3* was absent in Types 3 and 4 (Additional file 6: Fig. S5). Particularly, the *HD2.1* genes were grouped into three clusters with Type 1 and Type 3, whereas in *HD2.2*, Type 1 and Type 2 were grouped (Additional file 6: Fig. S6, S7).

**Fig. 3:**
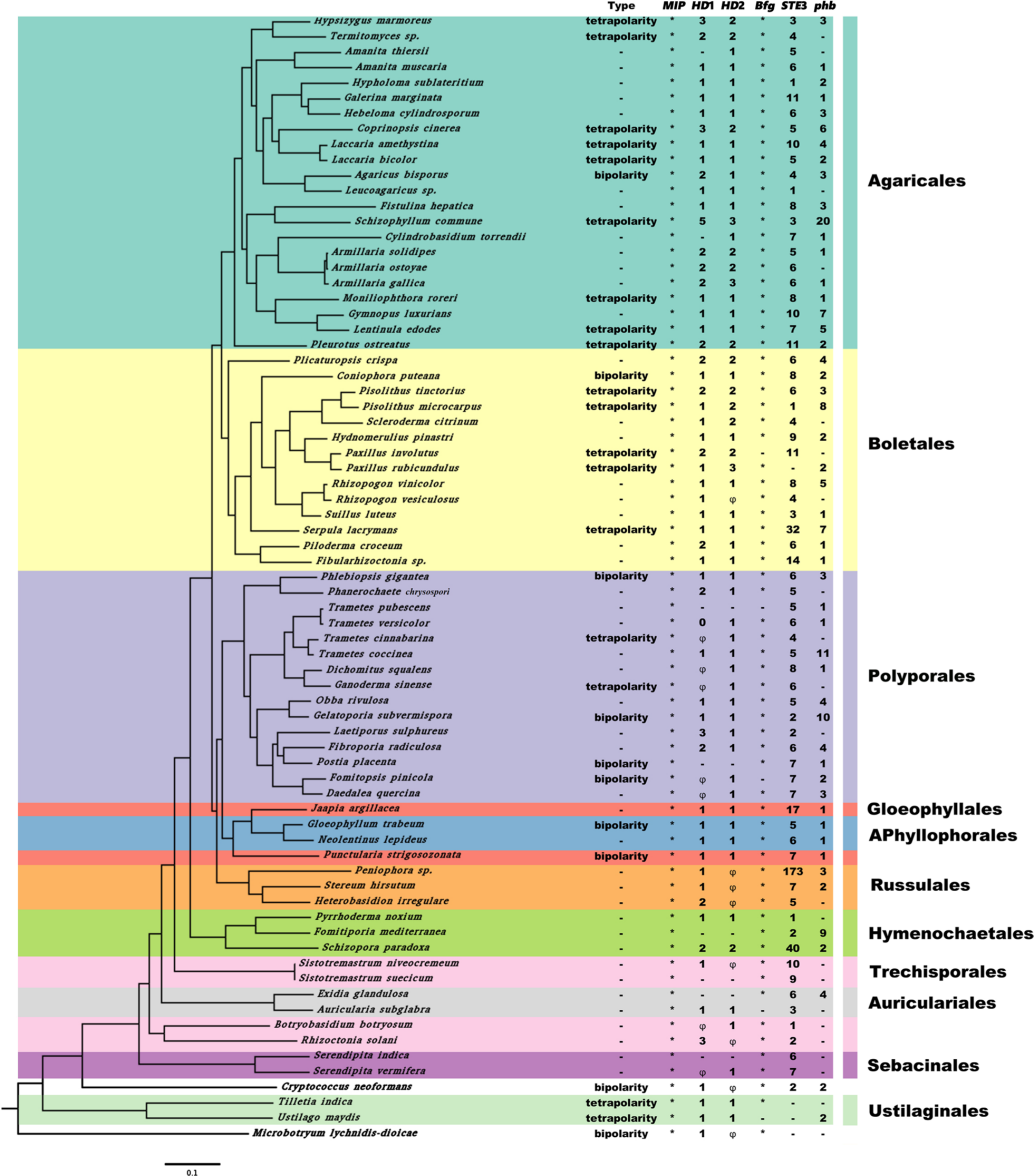
The phylogenetic trees of 73 fungal species from Basidiomycete species*.* The right one displays their mating system type and the number of following genes: *Mip*, *HD1*, *HD2*, *Bfg STE3* and *phb*. “φ” is representative of *MAT-2*, and "-" means the gene has not been detected

To investigate the evolution of mating-type loci in Basidiomycetes, we further identified *HD* genes from 73 different Basidiomycetes fungal species, Phylogenetic trees were constructed by single-copy homologous genes from the 73 fungal genomes (Fig. 3). The branches of Basidiomycetes were further divided into 11 subgroups, which corresponded to 11 orders as Agaricales, Boletales, Polyporales, Gloeophyllales, Aphyllophorales, Russulales, Hymenochaetales, Trechisporales, Auriculariales, Sebacinales, and Ustilaginales. We further identified these *HD* genes in the *HD* mating-type loci in the 73 Basidiomycete species fungal species. Basidiomycete species contained a single copy of *Mip* and *Bfg* (Fig. 3). Agaricales and its close order Boletales possessed *HD1* and *HD2*, whereas 37.8% of the examined species from other orders of Basidiomycetes*,* like Polyporales, Russulales, Hymenochaetales, Trechisporales, Auriculariales, and Sebacinales, only had one type of *HD1* or *HD2* (Fig. 3). In Basidiomycetes, each of these 11 orders contained tetrapolar and bipolar systems, indicating the common origin of these systems in fungi. HD proteins of Basidiomycete species were clearly divided into two clades (Additional file 6: Fig. S8), indicating that *HD1* and *HD2* originated before the origin of Basidiomycetes. Moreover, the phylogenetic trees based on *HD* genes from eight Agaricales fungi showed that three *HD1s* and two *HD2s* from each species were clustered together. This revealed that the *HD1s* and *HD2s* originated from tandem duplication after the specification of Agaricales (Additional file 6: Fig. S9).

### Collinearity analysis of the genomic regions for HD mating-type

**Fig. 4:**
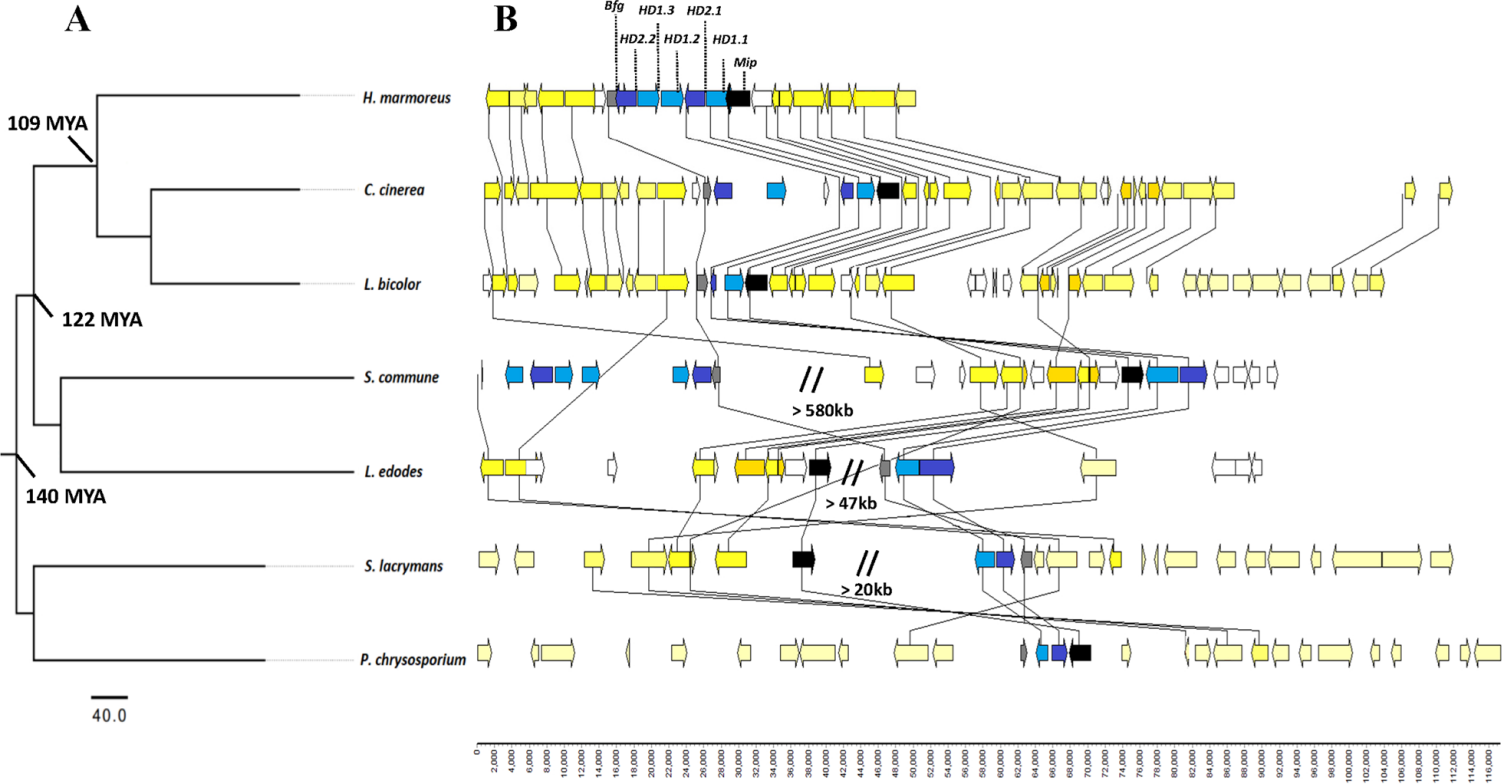
The phylogenetic trees of different strains of basidiomycete and collinearity analysis of genomic regions containing the *HD* mating type loci. The *Mip* and *Bfg* genes are displayed with black and brown arrows, respectively. *HD1* and *HD2* genes shown as sky blue and blue arrows, respectively. Yellow are representative other genes, non-mating *A* local genes

To unravel the events that altered the organization of HD mating-type region, we compared the synteny of genomic regions surrounding the *HD* loci of *H. marmoreus*, with that of six typical fungal species (Fig. 4). Consistent with the genetic relationship, a high level of synteny shared among *H. marmoreus*, *L. bicolor,* and *C. cinerea,* compared with other fungi, likely due to these three species being more closely related in the classification. In *HD* loci, all the *Mip* genes were neighboring *HD1*, except in *L. edodes* where the distance between the *Mip* and *A* loci was at least 47 kb apart and *S. lacrymans* (20 kb) with a chromosomal inversion, indicating that the *Mip-HD1* genomic regions were highly conserved for at least 140 million years (MYAs). In *S. commune*, a 580 kb genomic region was located on the chromosomes between *HD* gene pairs, whereas in *C. cinerea*, these two *HD* gene pairs were separated by a putative gene. These results indicated that chromosome recombination and gene insertion may cause the different mechanisms for the evolution of HD mating-type in Basidiomycetous (Kämper et al. 2020).

### The genome structure of *P/R* mating-type loci in *H. marmoreus* strains

**Fig. 5:**
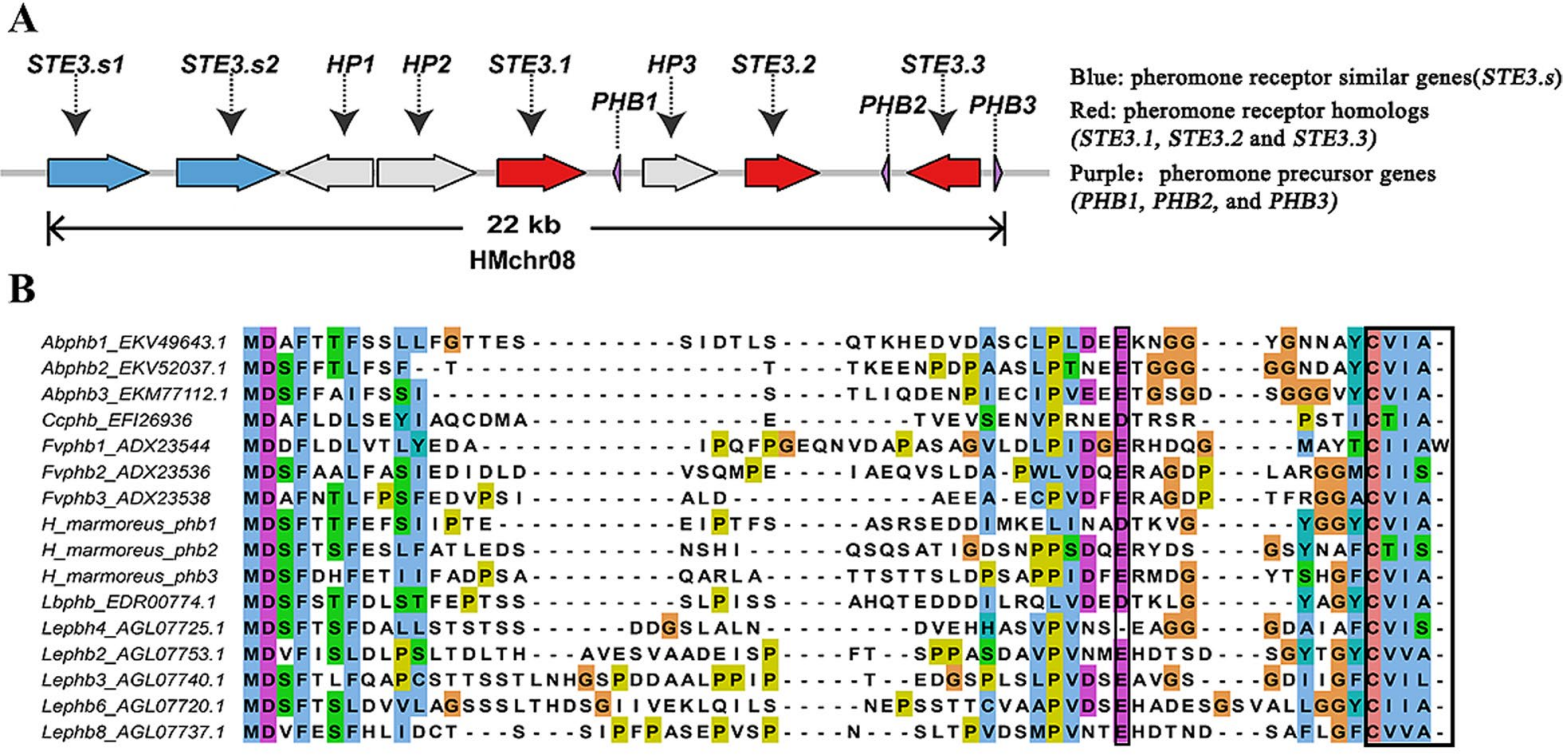
The mating *P/R* loci structure and pheromone precursor protein. **A** The mating *P/R* loci structures. The gene names shown above each arrow. **B** Alignment of the pheromone precursor amino acid sequences from *H. marmoreus*, *C. cinerea* (Cc), *L. bicolor* (Lb), *A. bisporus* (Ab), *L. edodes* (Le), and *F. velutipes* (Fv), showing typical conservation of the CAAX motifs, and a Glu (E) at a position 10 AA upstream from the CAAX motif, which are both needed for proper maturation

*P/R* loci are typically composed of pheromone receptor and pheromone precursor genes. Based on pheromone receptor protein from *C. cinerea*, *S. commune,* and *L. edodes* as queries, a 22 kb genomic region on Chr08 of *H. marmoreus* was identified as the P/R mating-type. The genomic region harbored three pheromone receptor homologs (*STE3.1*, *STE3.2,* and *STE3.3*), two pheromone receptor similar genes (*STE3.s1* and *STE3.s2*)*,* three pheromone precursor genes (*PHB1*, *PHB2,* and *PHB3*), and three hypothetical protein genes (*HP1*, *HP2* and *HP3*) (Fig. 5A). These three *PHBs* encoded a protein around 50 AA in length and contained a CAAX motif in the C-terminus (Kim et al. 2014). *PHB1* and *PHB3* had a CVIA motif, whereas *PHB2* contained a CTIS motif. *PHB3* and *PHB 2* were located on two sides of *STE3.3,* with a CVIA motif. A typical conserved glutamic acid (Glu, “E”) was located on 10 AA upstream of the C-terminal CAAX box. Similar structural characteristics of the genome were also existed in other species of tetrapolar Basidiomycetes*,* including *C. cinerea*, *L. bicolor*, *A. bisporus*, *L. edodes,* and *F. velutipes* (Fig. 5B).

### Phylogenetic analysis, gene structure, and motif composition of the pheromone receptors

**Fig. 6:**
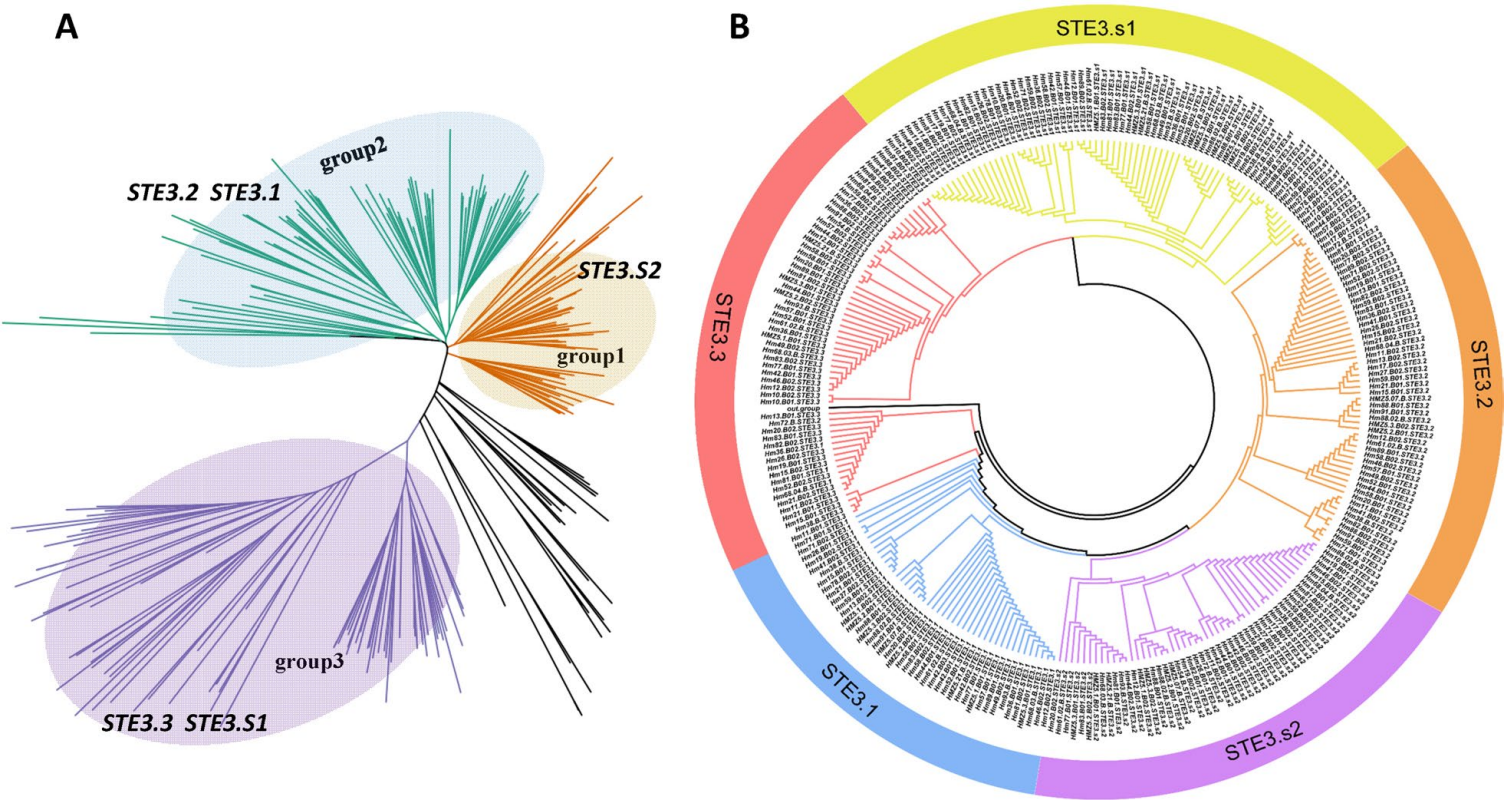
Phylogenetic tree depicting the relationship between pheromone receptor and pheromone receptor-like proteins. **A** Interspecific evolutionary tree between pheromone receptor and pheromone receptor-like proteins of Basidiomycete species. *STE3.1*, *STE3.2* and *STE3.s2* clustered in one branch (group 1 and group 2), *STE3.3* and *STE3.s1* were in another (group 3). **B** Intraspecific evolutionary tree between pheromone receptor and pheromone receptor-like proteins of *H. marmoreus*

We annotated the P/R mating-type genomic regions of 54 *H. marmoreus* strains. Forty-nine *STE3.1*, 55 *STE3.2*, 53 *STE3.3*, 60 *STE3.s1, and* 48 *STE3.s2* were identified in the genomic regions of population. These genes were further used to construct phylogenetic trees, which clearly showed that *STE3.1*, *STE3.2*, and *STE3.s2* were distributed in one clade, and *STE3.3* and *STE3.s1* were in another (Fig. 6B, Additional file 6: Fig. S10-S12). These results suggested that *STE3.1*/*STE3.2*/*STE3.s2* and *STE3.3/STE3.s1* independently originated from gene tandem duplication events. The exon–intron organizations of *STE3* and *STE3.s* genes from the 54 strains were examined to gain further insight into the evolution of *P/R* mating-type genes in *H. marmoreus*. The pheromone receptor genes within the same group generally had a similar structure, and *STE* genes were clustered together, except *STE3.3* as shown in Additional file 6: Fig. S13C. The three *STEs* genes such as*STE3.1*, *STE3.2*, and *STE3.s2* were possessed five introns, *STE3.3* and *STE3.s1* possessed four introns, excluding the haplotypes from three strains (*HMZ5.9.B.STE3.3*, *HM88.W2B.STE3.3*, and *HMZ.07.B.STE3.3)* with five introns (Additional file 6: Fig. S13). The main difference between the genes in these two branches was the number of introns in *STE* domains, indicating functional differentiation of these two sets of pheromone receptor genes. A schematic representing the structure of all *STE* proteins was constructed from the motif analysis results. As shown in Additional file 6: Fig. S13B, *STE3* members within the same groups generally shared a similar motif composition, except *HMZ5_9_STE3.3, HM88_2_STE3.3,* and *HMZ_07_STE3.3,* suggesting that *STE3.3* proteins were highly polymorphic. Similar motif arrangements among *STE3* subgroups indicated that the protein architecture was conserved within a specific pheromone receptor. Overall, the conserved motif compositions and similar gene structures of *STE3* members in the same group, together with the phylogenetic analysis results, strongly supported the reliability of the group classifications.

We searched *P/R* loci from genome of 73 fungal species of Basidiomycetes by using the Perl script and found that the pheromone receptor *STE3* genes were existed in all Basidiomycete species. *STE3.1*, *STE3.2,* and *STE3.s2* were clustered into a single branch (Group 1 and Group 2), and *STE3.3* and *STE3.s1* were clustered into another (Group 3). In the interspecific strains of Agaricales, three distinct clades were grouped with basal *STE3* and *STE3.s* (Additional file 6: Fig. S14). The *STE3* and *STE3.s* genes from different species were clustered together, *STE3.s1* and *STE3.s2* in *H. marmoreus* and *Termitomyces.* spp were more closely related to each other, indicating that all *STE3* and *STE3.s* have the primordial genes that originated before the formation of Agaricales*.* The pheromone gene was undetectable in some strains, likely due to its short amino acid sequence or low-quality genome assembly. In combination with the evolutionary tree constructed by *STE3* genes in these 73 fungi (Fig. 6, Additional file 6: Fig. S14), *STE3* genes had two main origins, among which *STE3.1* gene was copied from *STE3.2* before speciation. These pheromone receptors belonged to the out-paralog gene arose from a duplication event before the speciation event. In addition, we found that *STE3s* showed a deep-rooted polymorphism at the base of Agaricomycetes, similar to Trichosporonales and Tremellales (Sun et al. 2019), indicating that suppressed recombination for *STE3s* were not an independent event in the Agaricales, Trichosporonales*,* and Tremellales.

### Transcriptome analysis of karyogamy related genes in homokaryons and heterokaryons

**Fig. 7:**
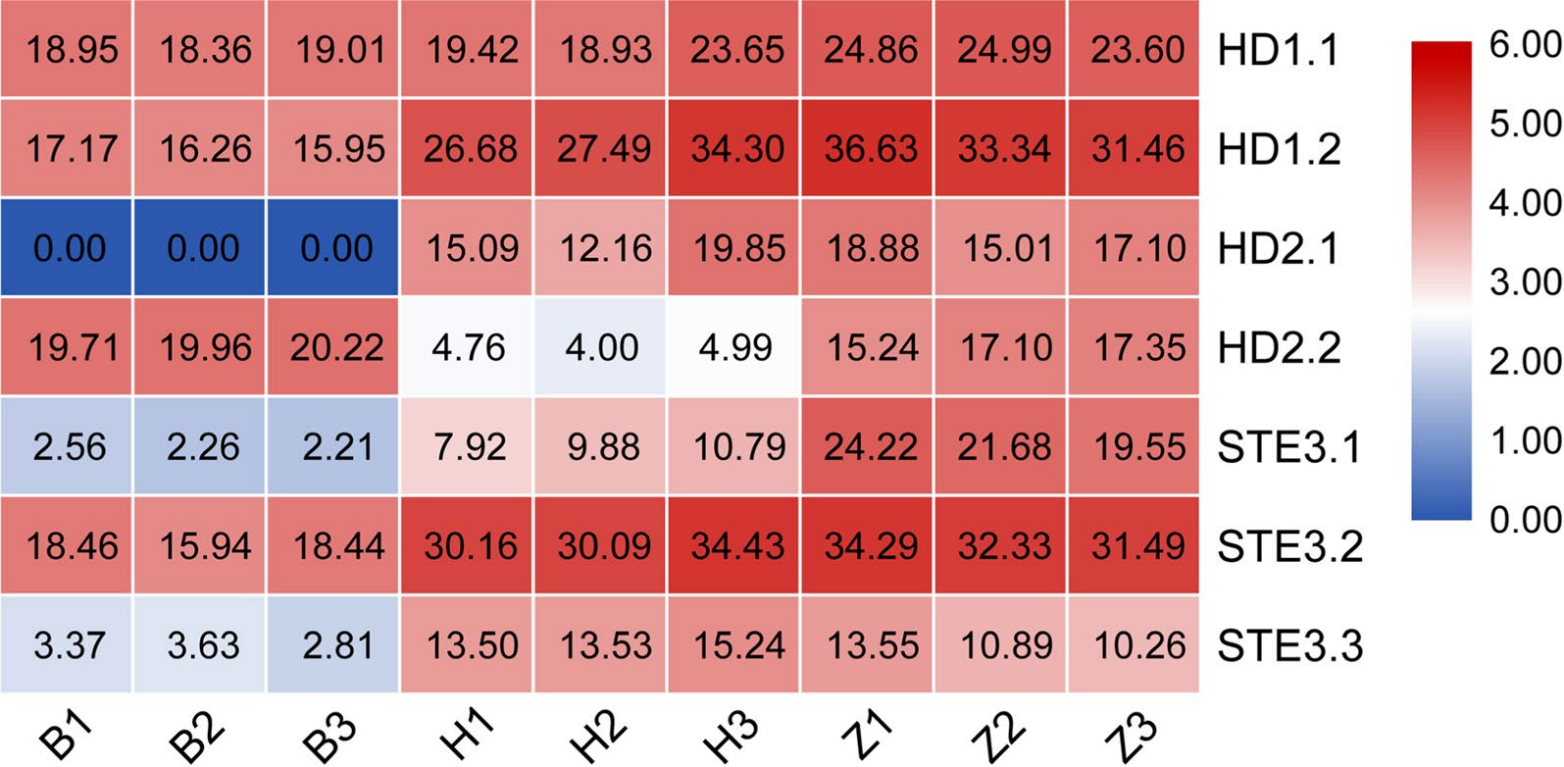
The expression of *HD* and *PR* mating-type genes in *H. marmoreus* strains Hm88-W2, Hm61-G6 and HMZ5. B indicates the monokaryotic strain Hm88-W2, H indicates the monokaryotic strain Hm61-G6, Zrepresents HMZ5, the hybrid strain of Hm88-W2 and Hm61-G6 and repeats three times. *HD*: homeodomain (HD) genes; *PR*: the pheromone receptor (STE)

To investigate the expression patterns of *HD* mating-type and *P/R* mating-type genes during the formation of dikaryotic mycelia by hybridization, three biological replications of Hm88-W2 and Hm61-G6 monokaryotic mycelium and their dikaryotic mycelium HMZ5 were collected for RNA-seq analysis. The overall reading alignment rate of the nine sequencing samples was higher than 70% (Additional file 5: Table S5). Both the correlation analysis and principal component analysis (PCA) of gene expression showed good reproducibility and high similarity (Additional file 6: Fig. S15, S16). Based on the false discovery rate (FDR) ≤ 0.05 and |log_2_FC|≥ 1, 2494, 2059, and 2272 differentially expressed genes (DEGs) were identified from Hm88-W2/Hm61-G6, Hm88-W2/HMZ5, and Hm61-G6/HMZ5, respectively (Additional file 6: Fig. S17). Further analysis of Hm88-W2, Hm61-G and Hm62-W as reference genomes revealed the differential expression of *HD* mating-type and pheromone receptor genes. With Hm88-W2 as reference genome, *HD1.1*, *HD1.2*, *HD1.3*, *STE3.2,* and *STE3.3* were expressed only in Hm88-W2 and HMZ5. When Hm61-G6 was used as reference genome, the results were vice versa. With Hm62-W as reference genome, *HD1.1* and *HD1.2* were expressed at low levels, and *STE3.1*, *SEE3.2,* and *STE3.3* were expressed in Hm61-G6 and HMZ5 (Additional file 6: Fig. S18). These results may be related to reference sequences based on differences of alleles. Combined with the expression of *MAT* genes, *STE3.1* was differentially expressed among Hm88-W2, Hm61-G, and HMZ5, suggesting that *STE3.1* play an important role in fruiting process (Fig. 7).

GO functional enrichment analysis of DEGs in Hm88-W2 *v.s.* HMZ5 and Hm61-G6 *v.s.* HMZ5 revealed that these genes were mainly involved in “metabolic process,” “integral component of membrane,” “intrinsic component of membrane,” “membrane,” “membrane part,” “catalytic activity,” “oxidoreductase activity,” and “oxidation–reduction process” (Additional file 6: Fig. S19, S20). KEGG analysis (*p* ≤ 0.05) showed that 60 pathways were enriched in DEGs in Hm88-W2 versus HMZ5 and Hm61-G6 versus HMZ5. Compared with Hm88-W2 and Hm61-G6, upregulated genes in HMZ5 were mainly enriched in “calcium signaling pathway,” “melanin,” “mitogen-activated protein kinase (MAPK) signaling pathway,” and “serine and threonine metabolism” (Additional file 6: Fig. S21). Notably, 93 genes including *STE3.1*, *STE3.2*, *STE3.3 STEs1,* and *STEs2* were enriched in the MAPK pathway, indicating that MAPK signaling may be important during *H. marmoreus* mating.

## DISCUSSION

Mushrooms possess thousands of distinct mating types (Casselton and Olesnicky 1998). They usually have two pairs of divergently transcribed *HD* genes in outward transcriptional directions and an additional *HD1* gene in the middle of the groups, as identified first in the *H. marmoreus* genome. Like a few Basidiomycetes such as *C. cinerea* and *S. commune, H. marmoreus* possess multiple *HD* genes (Rong et al. 2015; Mujic et al. 2017). Our results suggested that in tetrapolar Basidiomycete strains, a pair of *HD* genes act as a dominant HD mating-type genome structure, and that multiple alleles of *MAT* genes may arise through the evolution of distinct DNA sequences. Of the five *HD* genes (*HD1.1, HD1.2, HD1.3, HD2.1,* and *HD2.2*) in *H. marmoreus, HD1.1*, *HD1.2*, and *HD2.2* were present in all *H. marmoreus* strains, whereas *HD1.3* was absent in 24 haplotypes and *HD2.1* was absent in 22 haplotypes. The analysis of genomic regions of HD mating-type supported that *HD2.1* and *HD1.3* are not the key transcriptional factors for mating compatibility since Type 2 (absence of *HD2.1*), Type 3 (absence of *HD1.3*), and Type 4 (absence of *HD2.1* and *HD1.3*) were detected in 54 monokaryon/dikaryon strains. Each HD heterodimerizes with a partner encoded by the genes in the same type (group) as seen in Table 1 in dikaryotic strains Hm13, Hm15, Hm17, Hm71, Hm78 which are Type 1 & Type 1 (Additional file 1: Table S1), but fails to heterodimerize with a partner (gene product encoded) in the same loci (Brown and Casselton 2001). In addition, the four types of *HD* loci (haplotype) were conserved, heritable, and interlinked. This provided theoretical support for the relative conserved of the mating types of each strain of Basidiomycetes*.*

**Table 1:** Dikaryotic strains of *H. marmoreus* containing different HD mating-types types

Type	Strains
Type 1 & Type 1	Hm13, Hm15, Hm17, Hm71, Hm78
Type 1 & Type 2	Hm20, Hm47, Hm46, Hm62, Hm77, Hm81, Hm83, Hm89
Type 1 & Type 3	Hm3, Hm10, Hm11, Hm12, Hm16, Hm19, Hm26, Hm30, Hm38, Hm56, Hm59, Hm72, Hm82, Hm42, Hm41
Type 2 & Type 3	Hm60, Hm9, Hmz9
Type 2 & Type 4	Hmz2, Hm12, Hm44, Hm53, Hm57, Hm88, Hm91
Type 3 & Type 4	Hmz5, Hmz1, Hmz6

In *H. marmoreus,* the duplicated genes, *HD1.3*, were undetectable for its expression level in both monokaryon and dikaryon. These results supported that the alleles of *HD1.3* were functional redundancies. Importantly, all the dikaryotic strains contained *HD1.1, HD1.2* and *HD2.2* in haploid /monokaryotic strains *HD* loci. These three *HD* genes were expressed in homokaryons and heterokaryons with dominant expression of *HD1.1* and *HD1.2*. Therefore, for mating compatibility, we assumed that *HD1.1*, *HD1.2,* and *HD2.2* are the key factors, whereas *HD1.3* and *HD2.1* were maybe essential but not sensitive to dosage effects in *H. marmoreus.* Consistent with the population analysis in *H. marmoreus*, our hybridization of two homokaryons HM61-G6 (Type 3) and HM88-W2 (Type 4) showed that Type 3 had mating compatibility with Type 4. However, of the four F2 basidiospores, HMZ5_07 (Type 4) only paired with HMZ5_21 (Type 3), and HMZ5_09 (Type 3) only paired with HMZ5_78 (Type 4). Comparative expression of *HD* loci genes in monokaryon and dikaryon mycelia revealed that *HD1.1*, *HD1.2,* and *HD2.2* expressed in monokaryon and dikaryon fungal states, but displayed a similar expression pattern among these examined samples, similar to *C. cinerea* (Richardson et al. 1993).

Phylogenetic trees of seven representative fungi in the current study supported a previously established classic taxonomic classification (Chen et al. 2016b). However, our study revealed that the genomes of HD mating-type were divergent in Basidiomycetes*,* even in the order Agaricales*,* generally conserved in these three close families including Lyophyllaceae (*H. marmoreus*), Psathyrellaceae (*C. cinerea*), and Tricholomataceae (*L. bicolor*). In comparison to *H. marmoreus*, segment insertion and gene translocation were observed in the HD mating-type genomic regions of *L. edodes* and *S. lacrymans*. The genome structure of *HD* loci indicated the conservation of the gene order (*Mip*-*HD1*-*HD2*-*Bfg*) in most Basidiomycetes*,* consistent with previous studies (James et al. 2004b; Niculita-Hirzel et al. 2008; Au et al. 2014). This was likely due to the limited fungal genome information available for those analyses. The divergence of *HD* loci among Agaricomycetes indicated that the evolution of mating patterns was multi-directional. *HD* genes were considered to be conserved in both tetrapolar and bipolar Basidiomycetes (Kües et al. 2011; Maia et al. 2015). Our results showed that the pairs of *HDs* (*HD1* and *HD2*) existed in most Basidiomycetes. These two dissimilar *HDs* were clearly divided into two clades, and species-specific gene duplication of *HDs* were common in Basidiomycetes*,* as shown by the phylogenetic tree that multiple *HDs* were clustered together from one fungal species. These results further indicated that HD1 and HD2 proteins being from the same sub-locus (haplotype) cannot heterodimerize, which is consistent with the study of Brown and Casselton (Brown and Casselton 2001). They also verified the previous hypothesis that *HD* genes were originated from the duplications of one original gene pair (Kües et al. 1992; Kamada 2002). Both Ascomycetes and Mucoromycotina orders are bipolar and they only harbor a mating-type locus (Wolfe and Butler 2017). Basidiomycetes contain tetrapolar and bipolar species, and the tetrapolar strain is considered to be the ancestor of the bipolar strain (Kües et al. 2011; Maia et al. 2015). Our results suggested that the bipolar strains may be evolved from gene fusion events or losses in tetrapolar in the mating-type loci (Sun et al. 2019).

In this study, the majority (79.5%) of the analyzed Basidiomycete species harbored at least two *HD* genes in mating-type loci. Only one type of *HD* gene (*HD1s* or *HD2s*) was present in both bipolar and tetrapolar mating systems of some species in five orders (Hymenochaetales, Auriculariales, Sebacinales, Russulales, and Trechisporales). These results indicated that the number of *HD* genes is not an indication for heterodimerization with each other from different proteins of a same sub-locus. One of the *HD* genes (*HD1s* or *HD2s*) played a decisive role in the dimerization process. A previous study suggested that the absence of one gene of *HD* pairs at *MAT* loci did not affect the mating function (Sun et al. 2019), and the species containing two different *HD* genes in *MAT* region were not able to properly pair during meiosis (Sun et al. 2019). So, we assumed that there are different dimerization models in Basidiomycete species. Maybe due to the gene duplication of *HD*, all species from both Agaricales and Boletales contained both *HD1* and *HD2* in the *HD* mating-type loci, explaining their advantageous hybridization. Multiple *HD* gene paralogs increased *HD* diversity within a species, and allowed more frequent dikaryon viability after mating with the existing *P/R* loci (Whittle and Johannesson 2011).

The *P/R* loci comprises pheromone receptors and pheromones, and each pheromone receptor is accompanied by one or several pheromones within a subloci in Basidiomycetes (Halsall et al. 2000; Raudaskoski and Kothe 2010). Here, three *STEs* (*STE3.1*, *STE3.2,* and *STE3.3*), three *PHBs* (*PHB1*, *PHB2,* and *PHB3*), and two *STEs* (*STE3.s1* and *STE3.s2*) were identified in a ~ 22 kb region on chromosome 8 of *H. marmoreus*. Similar to *Agrocybe salicacola* (Chen et al. 2017)*,* the pheromone gene in *H. marmoreus* were closely located to the pheromone receptor genes, indicating that it probably played a mating-type specific role as its orthologous gene. The *STEs* without neighboring *PHB* were not considered to participate in the mating-type discrimination (Chen et al. 2016a). In the present study, two *STEs* shared high similarity with pheromone receptor genes and were expressed in monokaryotic mycelium, heterokaryon mycelium, primordium, and fruiting bodies. The mitogen-activated protein kinase (MAPK) signaling pathway is widely conserved in eukaryotic organisms and is involved in the regulation of cell growth, differentiation, metabolism, and stress responses (Plotnikov et al. 2011). In our study, *STE3.1*, *STE3.2*, *STE3.3*, *STE.s1,* and *STE.s2* were enriched in the KEGG pathway and corresponding activation of the G protein-coupled MAPK cascade signaling pathway indicated that receptor genes and receptor-like genes play an important role in the growth and development of *H. marmoreus.* In addition, these two *STEs* contained four and three introns, respectively. They also belonged to the familiar G-protein-coupled receptor (GPCR) family with a classical seven transmembrane-spanning (7-TM) domain (Brown and Casselton 2001).

Further analysis revealed that *STE3.1*, *STE3.2,* and *STE3.s2* were distributed in one branch, whereas *STE3.3* and *STE3.s1* were distributed in multiple branches. The dikaryon HMZ5 was generated from the cross by monokaryon Hm88-W2 and Hm61-G6, and had vigorous hyphae, as shown by nuclear fusion and nuclear migration. *STE3.2* displayed similar expression levels between monokaryon and dikaryon stages. The expression of *STE3.1* was much higher at the dikaryon stage than that at the monokaryotic stage, and *STE3.3* was much lower expressed in HM88-W2 than in Hm61-G6 and HMZ5. These results were different from those of *P. eryngii*, and the transcription levels of *STE* genes were much higher in the monokaryon than in the dikaryon (Kim et al. 2014), suggesting the functional differential of *STE* between *P. eryngii* and *H. marmoreus,* and an important role of *STE3.1* during this process. The *P/R* loci regulates the migration of nuclei from one parental strain into another, the dissolution of the septum during nuclear migration within a foreign mycelium, and the fusion of *A*-induced clamp cells with the subapical hyphal cell (Kothe 1999; Au et al. 2014). Although the fungal monokaryon status required additional mating-specific pheromone and receptor genes for a monokaryon–monokaryon interaction to generate a dikaryon (Kim et al. 2014), our findings implied *STE3.1* and *STE3.2* were essential at the dikaryon rather than in the monokaryons, since the dosage compensation existed in the dikaryon in the expression pattern of *STE3.1* and *STE3.2*.

## CONCLUSIONS

In this study, we analyzed the haplotypes of two unlinked MAT loci (*HD* and *P/R*) in a *H. marmoreus* population, and performed comparative genomics analysis of these MAT loci for 73 representative Basidiomycetes species. Our results revealed that, within the *MAT* loci in *H. marmoreus*, the gene orders were conserved, while the gene contents were variable. In *H. marmoreus*, the *HD* loci haplotypes were further classified into four types and displayed heritable and tightly linked at the *HD* loci. The *P/R* locus genes contains three pheromone receptors, three pheromones, and two pheromone receptor-like genes. Transcriptome dynamics suggested that both the *HD* and *P/R* loci acted as switches for mating processes in *H. marmoreus*. Phylogenetic analyses of *P/R* loci genes for 73 representative *Basidiomycetes* revealed that the *STE3* genes differentiated prior to speciation. The diversity of *HD* and *P/R* loci in Agaricales and Boletales likely contribute to the mating compatibility. Further collinear analysis of *HD* loci suggested that the evolution of physically linked *MAT* loci in *C. cinerea* and *S. commune* have undergone different mechanisms. Our study provided insights into the evolutionary diversity of mating-type (*MAT*) loci in *H.* *marmoreus* and Basidiomycetes.

## Supplementary Information

**Additional file 1: Table S1.** Source of *Hypsizigus marmoreus* strains.

**Additional file 2: Table S2.** The fungi data source from NCBI.

**Additional file 3: Table S3.** The length of *MAT-A* genes among different haplotypes.

**Additional file 4: Table S4.** The length of each gene in the mating *A* locus.

**Additional file 5: Table S5.** The ratio of clean reads in the samples of true *H. marmoreus.*

**Additional file 6: Fig. S1.** Collinearity analysis of the *MAT-A* locus between the 6 strains of *H. marmoreus.* **Fig. S2.** The HD mating-type loci structure of 15 *H. marmoreus.* **Fig. S3.** Phylogenetic tree of *HD1.1* of *H. marmoreus* stains. The Ty1, Ty2, Ty3 and Ty4 represents haplotypes of *MAT-A* locus, respectively. **Fig. S4.** Phylogenetic tree of *HD1.2* of *H. marmoreus* stains. The Ty1, Ty2, Ty3 and Ty4 represents haplotypes of *MAT-A* locus, respectively. **Fig. S5.** Phylogenetic tree of *HD1.3* of *H. marmoreus* stains. The Ty1, Ty2, Ty3 and Ty4 represents haplotypes of *MAT-A* locus, respectively. **Fig. S6.** Phylogenetic tree of *HD2.1* of *H. marmoreus* stains. The Ty1, Ty2, Ty3 and Ty4 represents haplotypes of *MAT-A* locus, respectively. **Fig. S7.** Phylogenetic tree of *HD2.2* of *H. marmoreus* stains. The Ty1, Ty2, Ty3 and Ty4 represents haplotypes of *MAT-A* locus, respectively. **Fig. S8.** Phylogenetic tree of *HD* genes between basidiomycetes. **Fig. S9.** Phylogenetic tree of *HD* among 8 Agaricales species. **Fig. S10.** Phylogenetic tree of Pheromone receptor *STE3.1* from all different *H. marmoreus* stains. The B1and B2 represents the allele of the heteronuclear strain, respectively. **Fig. S11.** Phylogenetic tree of Pheromone receptor *STE3.2* from all different *H. marmoreus* stains. The B1and B2 represents the allele of the heteronuclear strain, respectively. **Fig. S12.** Phylogenetic tree of Pheromone receptor *STE3.3* from all different *H. marmoreus* stains. The B1and B2 represents the allele of the heteronuclear strain, respectively. **Fig. S13.** Phylogenetic relationships, gene structure and architecture of conserved protein motifs in *STE3* genes from the different strains. (A). The phylogenetic tree was constructed based on the full-length sequences of *SET3* proteins. Details of clusters are shown in different colors. (B). The motif composition of *STE3* proteins. The motifs, numbers 1–15, are displayed in different colored boxes. (C). Exon–intron structure of *STE3* genes. Blue boxes indicate untranslated 5’- and 3’-regions; yellow boxes indicate exons; black lines indicate introns. The *STE3* domains are highlighted by pink boxes. The number indicates the phases of corresponding introns. The length of protein can be estimated using the scale at the bottom. **Fig. S14.** Phylogenetic tree of *STE* genes of *Agaricales* species. The *STE3* genes display the deep-rooted trans-species polymorphism. **Fig. S15.** Matrix heat map of Pearson’s correlation coefficients for pairs from nine samples of *H. marmoreus*. The color represents the degree of correlation between repetitions in the sample. B indicates the mononuclear strain Hm88-W2, H indicates the mononuclear strain Hm61-G6, Z represents HMZ5, the hybrid strain of Hm88-W2and Hm61-G6. **Fig. S16.** PCA analysis of the gene expression levels for RNA-seq samples from two mononuclear stains and their hybrid stain*.* B indicates the mononuclear strain Hm88-W2, H indicates the mononuclear strain Hm61-G6, Z represents HMZ5, the hybrid strain of Hm88-W2and Hm61-G6. **Fig. S17.** Comparative analysis of differentially expressed genes in mononuclear and binuclear mycelium stages. (A). Venn diagram showing the number of detected proteins overlapping among three samples. (B). Red and blue numbers denote the numbers of significantly upregulated and downregulated gene respectively. **Fig. S18.** The gene expression were analyzed by reference genomes of different strains. B indicates the mononuclear strain Hm88-W2, H indicates the mononuclear strain Hm61-G6, Z represents HMZ5, the hybrid strain of Hm88-W2and Hm61-G6. **Fig. S19.** GO enrichment bubble map of down-regulated gene of HMZ5/Hm61-G6. The vertical coordinate is the enrichment of the top 30 GO functions, and the horizontal coordinate is the Rich factor. P represents significance, and is represented by gradient from low to high (0 to 1). The lower the p value, the higher the GO enrichment degree. The number of genes enriched by GO in DEGs is expressed by bubble size. **Fig. S20.** GO enrichment bubble map of down-regulated gene of HMZ5/ Hm88-W2. GO enrichment bubble map of down-regulated gene of HMZ5/ Hm61-G6 stain. The vertical coordinate is the enrichment of the top 30 GO functions, and the horizontal coordinate is the Rich factor. P represents significance, and is represented by gradient from low to high (0 to 1). The lower the p value, the higher the GO enrichment degree. The number of genes enriched by GO in DEGs is expressed by bubble size. **Fig. S21.** KEGG enrichment bubble map of up-regulated gene of mononuclear/ heterocaryon.

## Data Availability

The genome sequences of *H. marmoreus* have been deposited at GeneBank in NCBI under the accession number of JABWDO000000000.1. The Sequence Read Archive (SRA) data of this study is available in NCBI GenBank under accession numbers: PRJNA508399, PRJNA644211, and PRJNA765720. The five *HD* (MT840826, MT840827, MT840828, MT840829, MT840830), three pheromone (MT840831, MT840832, MT840833), and five receptor (two like-receptor) gene sequences (MT840834, MT840835, MT840836, MT840837, MT840838) are available in NCBI GenBank with their accession numbers.

## Abbreviations

MAT: Mating-type loci
HD: Homeodomain
P/R: Pheromone/pheromone receptor genes
MIP: Mitochondrial intermediate peptidase
BFG: Beta-flanking gene
GPCRs: G protein-coupled receptors
PDA: Potato dextrose agar
SSIs: Single spore isolates
CCD_S_: Coiled-coil dimerization motifs
NLS: The nuclear localization signal
DEG_S_: Differentially expressed genes
FPKM: Fragments per kilobase of exon per million fragments mapped
FDR: False discovery rate
KEGG: Kyoto Encyclopedia of Genes and Genomes
GO: Gene Ontology
FIG: Figure

## Declarations

### Adherence to national and international regulations

The authors confirm that this manuscript respects the Nagoya Protocol to the Convention on Biological Diversity.
