# Supplementary material for: Genetic structure and evolutionary diversity of mating-type (MAT) loci in Hypsizygus marmoreus
Source: IMA Fungus. 2021 Dec 20;12:35. doi: 10.1186/s43008-021-00086-8 (PMC8686365; doi:10.1186/s43008-021-00086-8)
Supplement: Supplementary file 6 — Additional file 6: Fig. S1. Collinearity analysis of the MAT-A locus between the 6 strains of H. marmoreus. Fig. S2. The HD mating-type loci structure of 15 H. marmoreus. Fig. S3. Phylogenetic tree of HD1.1 of H. marmoreus stains. The Ty1, Ty2, Ty3 and Ty4 represents haplotypes of MAT-A locus, respectively. Fig. S4. Phylogenetic tree of HD1.2 of H. marmoreus stains. The Ty1, Ty2, Ty3 and Ty4 represents haplotypes of MAT-A locus, respectively. Fig. S5. Phylogenetic tree of HD1.3 of H. marmoreus stains. The Ty1, Ty2, Ty3 and Ty4 represents haplotypes of MAT-A locus, respectively. Fig. S6. Phylogenetic tree of HD2.1 of H. marmoreus stains. The Ty1, Ty2, Ty3 and Ty4 represents haplotypes of MAT-A locus, respectively. Fig. S7. Phylogenetic tree of HD2.2 of H. marmoreus stains. The Ty1, Ty2, Ty3 and Ty4 represents haplotypes of MAT-A locus, respectively. Fig. S8. Phylogenetic tree of HD genes between basidiomycetes. Fig. S9. Phylogenetic tree of HD among 8 Agaricales species. Fig. S10. Phylogenetic tree of Pheromone receptor STE3.1 from all different H. marmoreus stains. The B1and B2 represents the allele of the heteronuclear strain, respectively. Fig. S11. Phylogenetic tree of Pheromone receptor STE3.2 from all different H. marmoreus stains. The B1and B2 represents the allele of the heteronuclear strain, respectively. Fig. S12. Phylogenetic tree of Pheromone receptor STE3.3 from all different H. marmoreus stains. The B1and B2 represents the allele of the heteronuclear strain, respectively. Fig. S13. Phylogenetic relationships, gene structure and architecture of conserved protein motifs in STE3 genes from the different strains. (A). The phylogenetic tree was constructed based on the full-length sequences of SET3 proteins. Details of clusters are shown in different colors. (B). The motif composition of STE3 proteins. The motifs, numbers 1–15, are displayed in different colored boxes. (C). Exon–intron structure of STE3 genes. Blue boxes indicate untranslated 5’- and 3’-regi [file 43008_2021_86_MOESM6_ESM.pdf]

Supplemental Figures

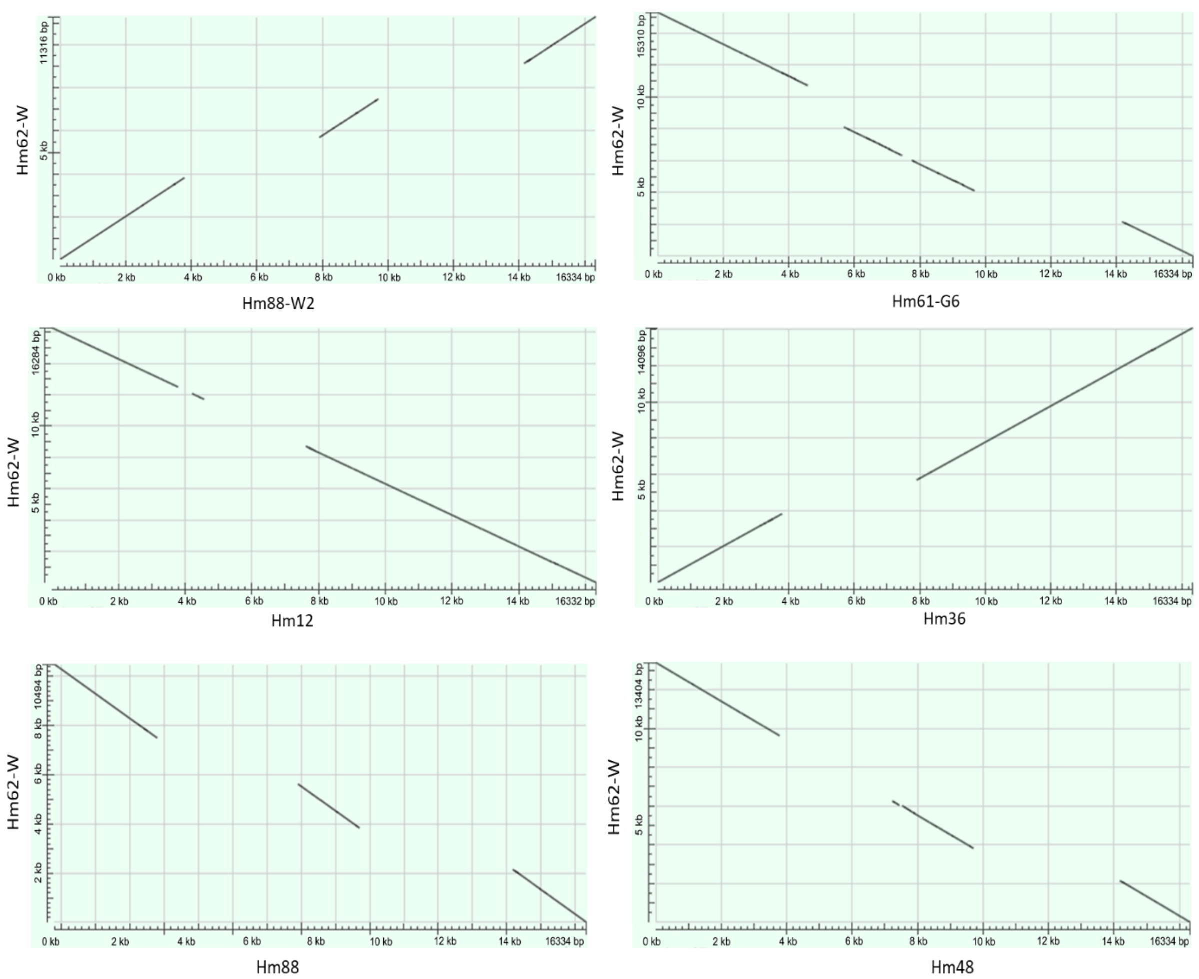

Figure S1. Collinearity analysis of the *MAT-A* locus between the 6 strains of *H. marmoreus.*

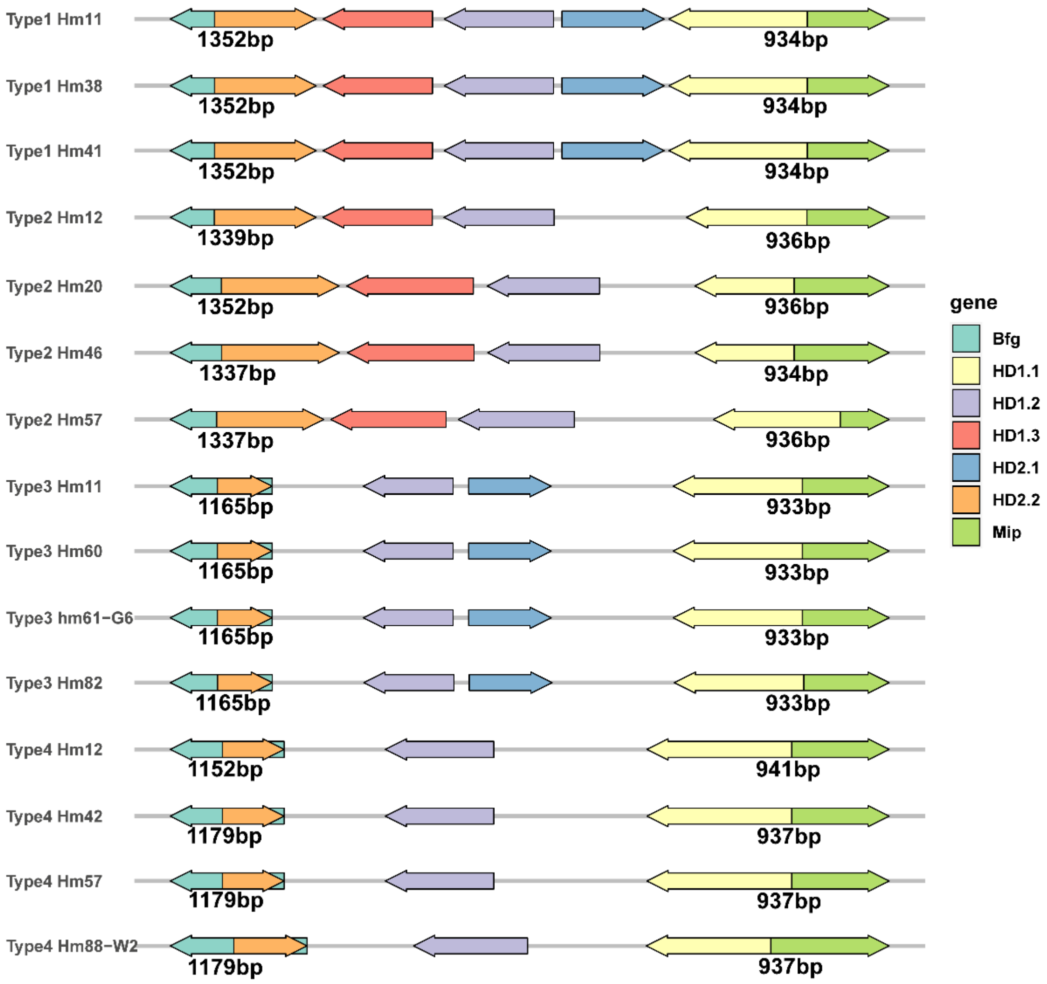

Figure S2. The HD mating-type loci structure of 15 *H. marmoreus*.

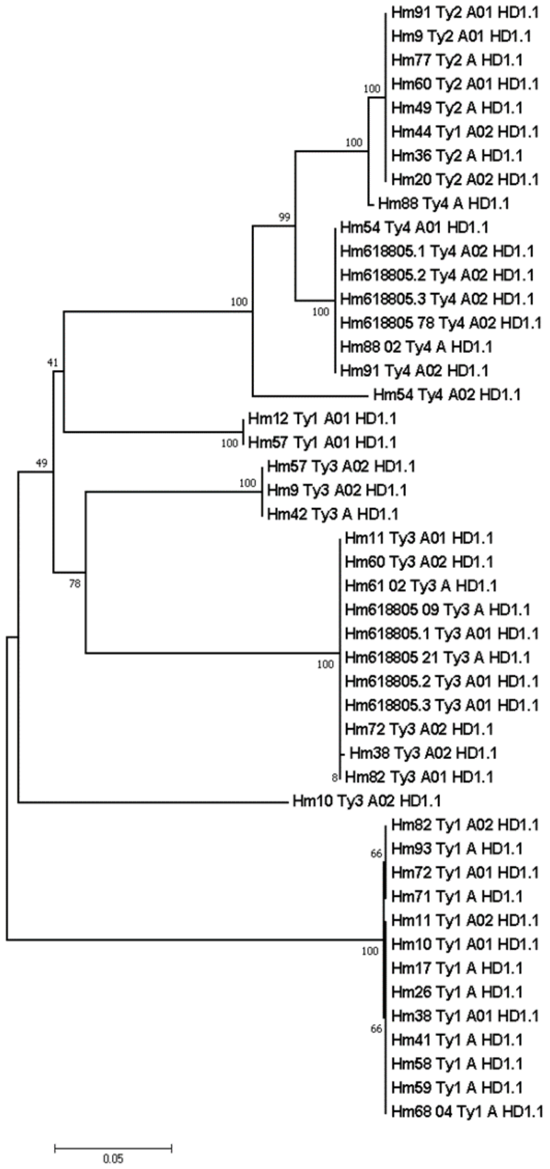

Figure S3. Phylogenetic tree of *HD1.1* of *H. marmoreus* stains. The Type1, Type2, Type3 and Type4 represents haplotypes of *MAT-A* locus, respectively.

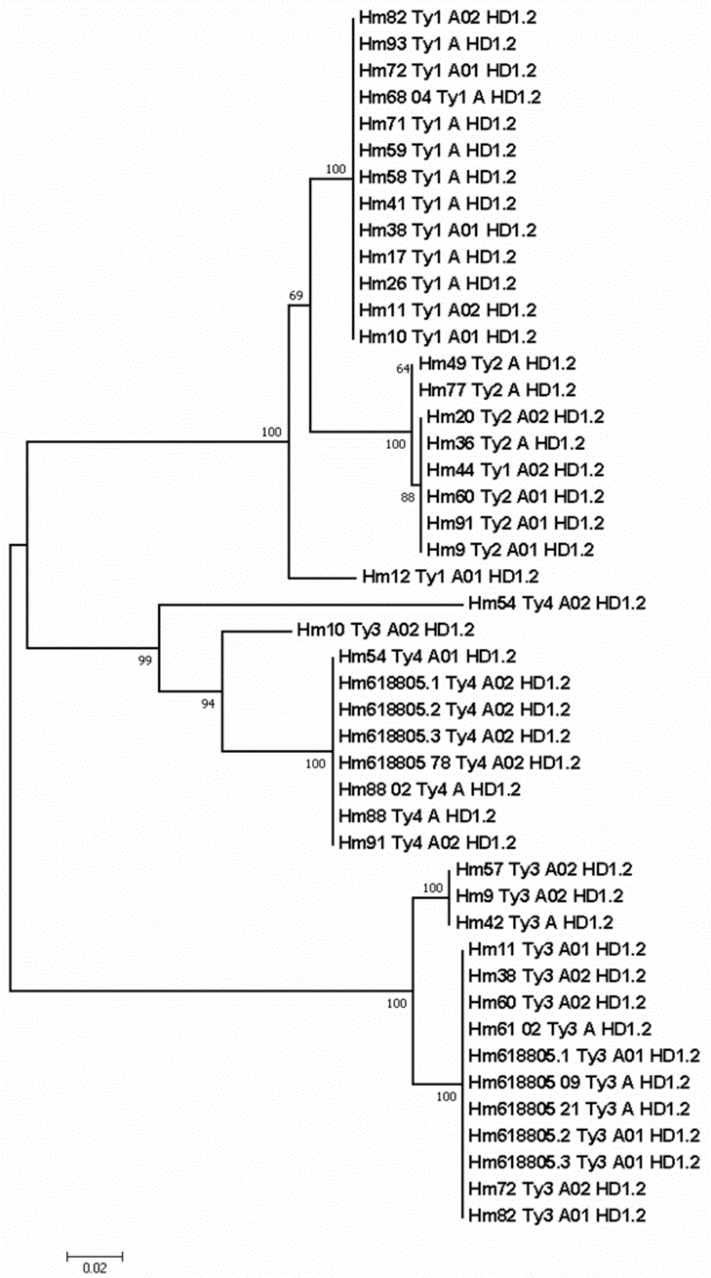

Figure S4. Phylogenetic tree of *HD1.2* of *H. marmoreus* stains. The Ty1, Ty2, Ty3 and Ty4 represents haplotypes of *MAT-A* locus, respectively.

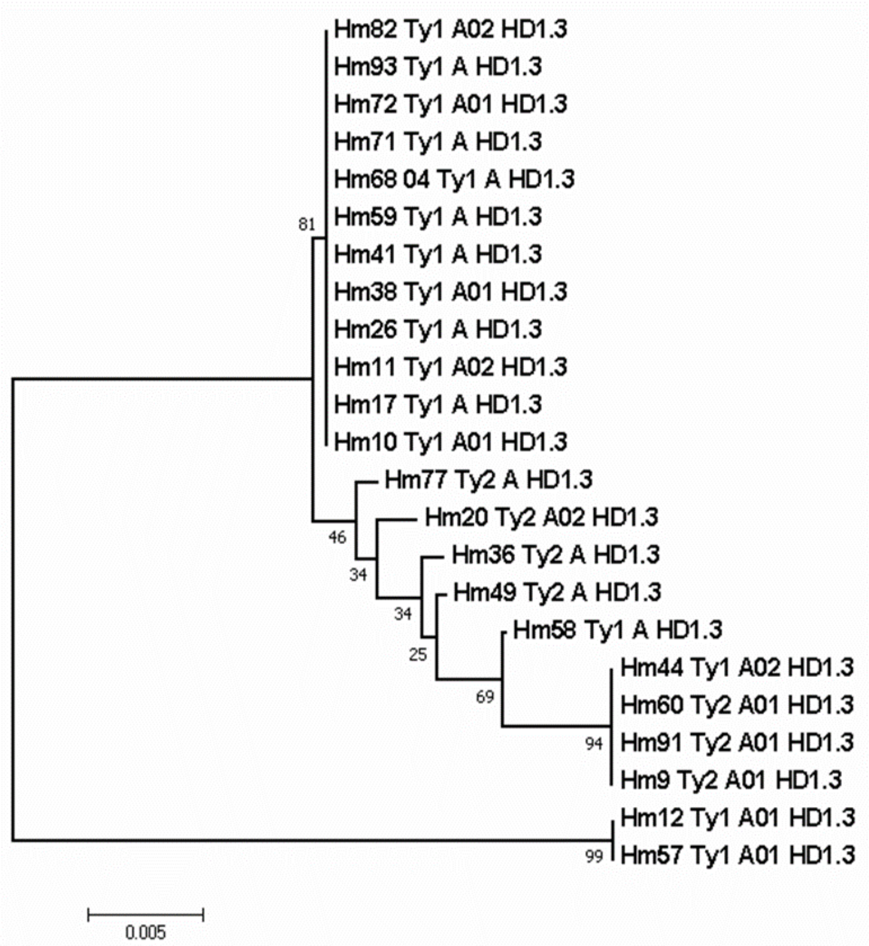

Figure S5. Phylogenetic tree of *HD1.3* of *H. marmoreus* stains. The Ty1, Ty2, Ty3 and Ty4 represents haplotypes of *MAT-A* locus, respectively.

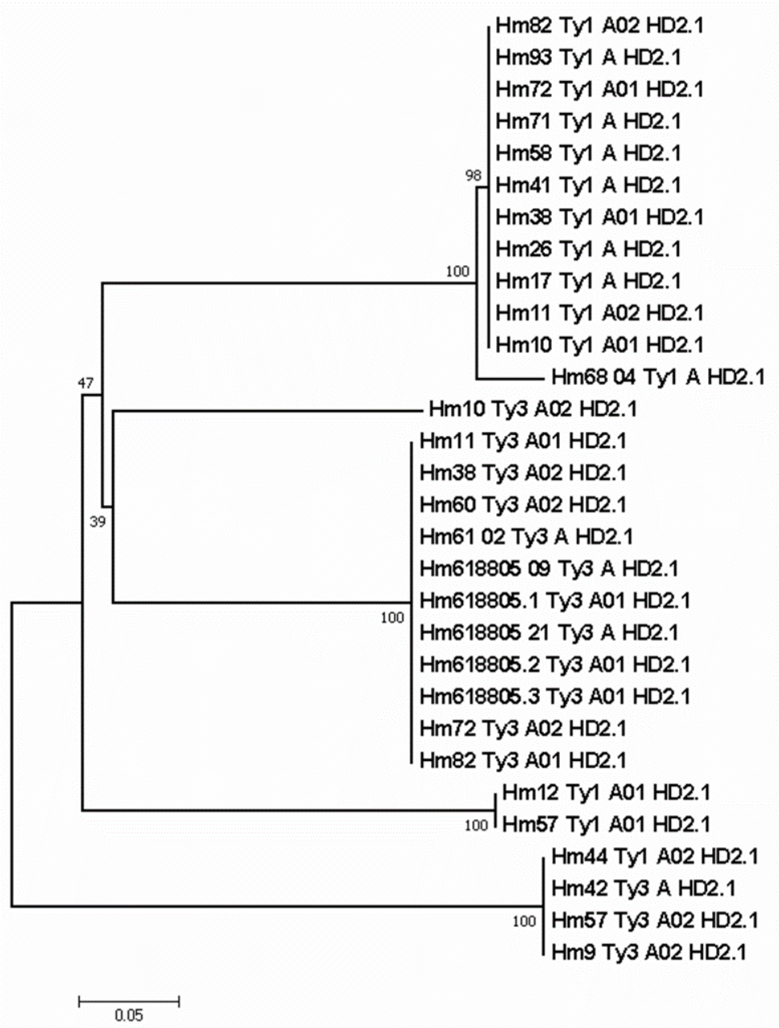

Figure S6. Phylogenetic tree of *HD2.1* of *H. marmoreus* stains. The Ty1, Ty2, Ty3 and Ty4 represents haplotypes of *MAT-A* locus, respectively.

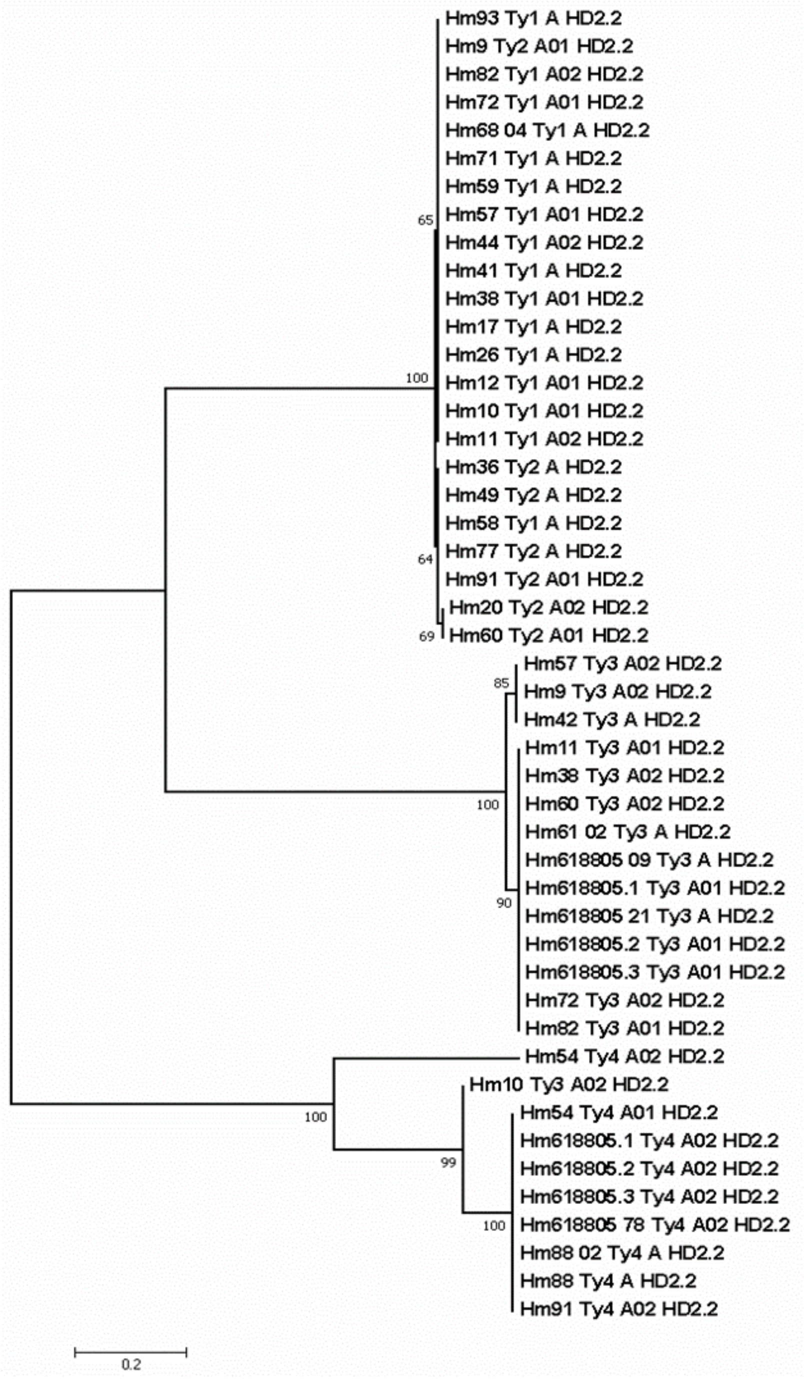

Figure S7. Phylogenetic tree of *HD2.2* of *H. marmoreus* stains. The Ty1, Ty2, Ty3 and Ty4 represents haplotypes of *MAT-A* locus, respectively.

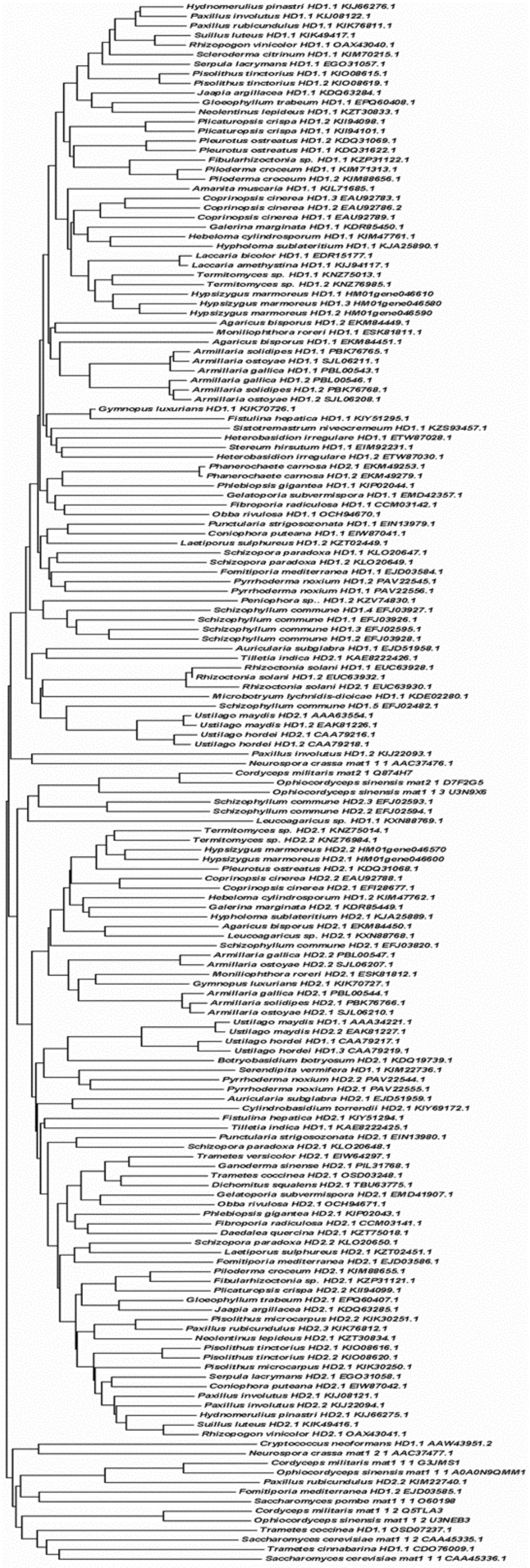

Hydnomerulius pinastri HD1.1 KIJ66276.1
Paxillus involutus HD1.1 KIJ08122.1
Paxillus rubicundulus HD1.1 KIK76811.1
Suillus luteus HD1.1 KIK49417.1
Rhizopogon vinicolor HD1.1 OAX43040.1
Scleroderma citrinum HD1.1 KIM70215.1
Serpula lacrymans HD1.1 EGO31057.1
Pisolithus tinctorius HD1.1 KIO08615.1
Pisolithus tinctorius HD1.2 KIO08619.1
Jaapia argillacea HD1.1 KDQ63284.1
Gloeophyllum trabeum HD1.1 EPQ60408.1
Neolentinus lepideus HD1.1 KZT30833.1
Plicaturopsis crispa HD1.2 KII94098.1
Plicaturopsis crispa HD1.1 KII94101.1
Pleurotus ostreatus HD1.2 KDQ31069.1
Pleurotus ostreatus HD1.1 KDQ31622.1
Fibularhizoctonia sp. HD1.1 KZP31122.1
Piloderma croceum HD1.1 KIM71313.1
Piloderma croceum HD1.2 KIM88656.1
Amanita muscaria HD1.1 KIL71685.1
Coprinopsis cinerea HD1.3 EAU92783.1
Coprinopsis cinerea HD1.2 EAU92786.2
Coprinopsis cinerea HD1.1 EAU92789.1
Galerina marginata HD1.1 KDR85450.1
Hebeloma cylindrosporum HD1.1 KIM47761.1
Hypholoma sublateritium HD1.1 KJA25890.1
Laccaria bicolor HD1.1 EDR15177.1
Laccaria amethystina HD1.1 KIJ94117.1
Termitomyces sp. HD1.1 KNZ75013.1
Termitomyces sp. HD1.2 KNZ76985.1
Hypsizygus marmoreus HD1.1 HM01gene046610
Hypsizygus marmoreus HD1.3 HM01gene046580
Hypsizygus marmoreus HD1.2 HM01gene046590
Agaricus bisporus HD1.2 EKM84449.1
Moniliophthora roreri HD1.1 ESK81811.1
Agaricus bisporus HD1.1 EKM84451.1
Armillaria solidipes HD1.1 PBK76765.1
Armillaria ostoyae HD1.1 SJL06211.1
Armillaria gallica HD1.1 PBL00543.1
Armillaria gallica HD1.2 PBL00546.1
Armillaria solidipes HD1.2 PBK76768.1
Armillaria ostoyae HD1.2 SJL06208.1
Gymnopus luxurians HD1.1 KIK70726.1
Fistulina hepatica HD1.1 KIY51295.1
Sistotremastrum niveocremeum HD1.1 KZS93457.1
Heterobasidion irregulare HD1.1 ETW87028.1
Stereum hirsutum HD1.1 EIM92231.1
Heterobasidion irregulare HD1.2 ETW87030.1
Phanerochaete carnosa HD2.1 EKM49253.1
Phanerochaete carnosa HD1.2 EKM49279.1
Phlebiopsis gigantea HD1.1 KIP02044.1
Gelatoporia subvermispora HD1.1 EMD42357.1
Fibroporia radiculosa HD1.1 CCM03142.1
Obba rivulosa HD1.1 OCH94670.1
Punctularia strigosozonata HD1.1 EIN13979.1
Coniophora puteana HD1.1 EIW87041.1
Laetiporus sulphureus HD1.2 KZT02449.1
Schizopora paradoxa HD1.1 KLO20647.1
Schizopora paradoxa HD1.2 KLO20649.1
Fomitiporia mediterranea HD1.1 EJD03584.1
Pyrrhoderma noxium HD1.2 PAV22545.1
Pyrrhoderma noxium HD1.1 PAV22556.1
Peniophora sp.. HD1.2 KZV74830.1
Schizophyllum commune HD1.4 EFJ03927.1
Schizophyllum commune HD1.1 EFJ03926.1
Schizophyllum commune HD1.3 EFJ02595.1
Schizophyllum commune HD1.2 EFJ03928.1
Auricularia subglabra HD1.1 EJD51958.1
Tilletia indica HD2.1 KAE8222426.1
Rhizoctonia solani HD1.1 EUC63928.1
Rhizoctonia solani HD1.2 EUC63932.1
Rhizoctonia solani HD2.1 EUC63930.1
Microbotryum lychnidis-dioicae HD1.1 KDE02280.1
Schizophyllum commune HD1.5 EFJ02482.1
Ustilago maydis HD2.1 AAA63554.1
Ustilago maydis HD1.2 EAK81226.1
Ustilago hordei HD2.1 CAA79216.1
Ustilago hordei HD1.2 CAA79218.1
Paxillus involutus HD1.2 KIJ22093.1
Neurospora crassa mat1 1 1 AAC37476.1
Cordyceps militaris mat2 1 Q874H7
Ophiocordyceps sinensis mat2 1 D7F2G5
Ophiocordyceps sinensis mat1 1 3 U3N9X6
Schizophyllum commune HD2.3 EFJ02593.1
Schizophyllum commune HD2.2 EFJ02594.1
Leucoagaricus sp. HD1.1 KXN88769.1
Termitomyces sp. HD2.1 KNZ75014.1
Termitomyces sp. HD2.2 KNZ76984.1
Hypsizygus marmoreus HD2.2 HM01gene046570
Hypsizygus marmoreus HD2.1 HM01gene046600
Pleurotus ostreatus HD2.1 KDQ31068.1
Coprinopsis cinerea HD2.2 EAU92788.1
Coprinopsis cinerea HD2.1 EFI28677.1
Hebeloma cylindrosporum HD1.2 KIM47762.1
Galerina marginata HD2.1 KDR85449.1
Hypholoma sublateritium HD2.1 KJA25889.1
Agaricus bisporus HD2.1 EKM84450.1
Leucoagaricus sp. HD2.1 KXN88768.1
Schizophyllum commune HD2.1 EFJ03820.1
Armillaria gallica HD2.2 PBL00547.1
Armillaria ostoyae HD2.2 SJL06207.1
Moniliophthora roreri HD2.1 ESK81812.1
Gymnopus luxurians HD2.1 KIK70727.1
Armillaria gallica HD2.1 PBL00544.1
Armillaria solidipes HD2.1 PBK76766.1
Armillaria ostoyae HD2.1 SJL06210.1
Ustilago maydis HD1.1 AAA34221.1
Ustilago maydis HD2.2 EAK81227.1
Ustilago hordei HD1.1 CAA79217.1
Ustilago hordei HD1.3 CAA79219.1
Botryobasidium botryosum HD2.1 KDQ19739.1
Serendipita vermifera HD1.1 KIM22736.1
Pyrrhoderma noxium HD2.2 PAV22544.1
Pyrrhoderma noxium HD2.1 PAV22555.1
Auricularia subglabra HD2.1 EJD51959.1
Cylindrobasidium torrendii HD2.1 KIY69172.1
Fistulina hepatica HD2.1 KIY51294.1
Tilletia indica HD1.1 KAE8222425.1
Punctularia strigosozonata HD2.1 EIN13980.1
Schizopora paradoxa HD2.1 KLO20648.1
Trametes versicolor HD2.1 EIW64297.1
Ganoderma sinense HD2.1 PIL31768.1
Trametes coccinea HD2.1 OSD03248.1
Dichomitus squalens HD2.1 TBU63775.1
Gelatoporia subvermispora HD2.1 EMD41907.1
Obba rivulosa HD2.1 OCH94671.1
Phlebiopsis gigantea HD2.1 KIP02043.1
Fibroporia radiculosa HD2.1 CCM03141.1
Daedalea quercina HD2.1 KZT75018.1
Schizopora paradoxa HD2.2 KLO20650.1
Laetiporus sulphureus HD2.1 KZT02451.1
Fomitiporia mediterranea HD2.1 EJD03586.1
Piloderma croceum HD2.1 KIM88655.1
Fibularhizoctonia sp. HD2.1 KZP31121.1
Plicaturopsis crispa HD2.2 KII94099.1
Gloeophyllum trabeum HD2.1 EPQ60407.1
Jaapia argillacea HD2.1 KDQ63285.1
Pisolithus microcarpus HD2.2 KIK30251.1
Paxillus rubicundulus HD2.3 KIK76812.1
Neolentinus lepideus HD2.1 KZT30834.1
Pisolithus tinctorius HD2.1 KIO08616.1
Pisolithus tinctorius HD2.2 KIO08620.1
Pisolithus microcarpus HD2.1 KIK30250.1
Serpula lacrymans HD2.1 EGO31058.1
Coniophora puteana HD2.1 EIW87042.1
Paxillus involutus HD2.1 KIJ08121.1
Paxillus involutus HD2.2 KIJ22094.1
Hydnomerulius pinastri HD2.1 KIJ66275.1
Suillus luteus HD2.1 KIK49416.1
Rhizopogon vinicolor HD2.1 OAX43041.1
Cryptococcus neoformans HD1.1 AAW43951.2
Neurospora crassa mat1 2 1 AAC37477.1
Cordyceps militaris mat1 1 1 G3JMS1
Ophiocordyceps sinensis mat1 1 1 A0A0N9QMM1
Paxillus rubicundulus HD2.2 KIM22740.1
Fomitiporia mediterranea HD1.2 EJD03585.1
Saccharomyces pombe mat1 1 1 O60198
Cordyceps militaris mat1 1 2 Q5TLA3
Ophiocordyceps sinensis mat1 1 2 U3NEB3
Trametes coccinea HD1.1 OSD07237.1
Saccharomyces cerevisiae mat1 1 2 CAA45335.1
Trametes cinnabarina HD1.1 CDO76009.1
Saccharomyces cerevisiae mat1 1 1 CAA45336.1

Figure S8. Phylogenetic tree of *HD* genes between basidiomycetes.

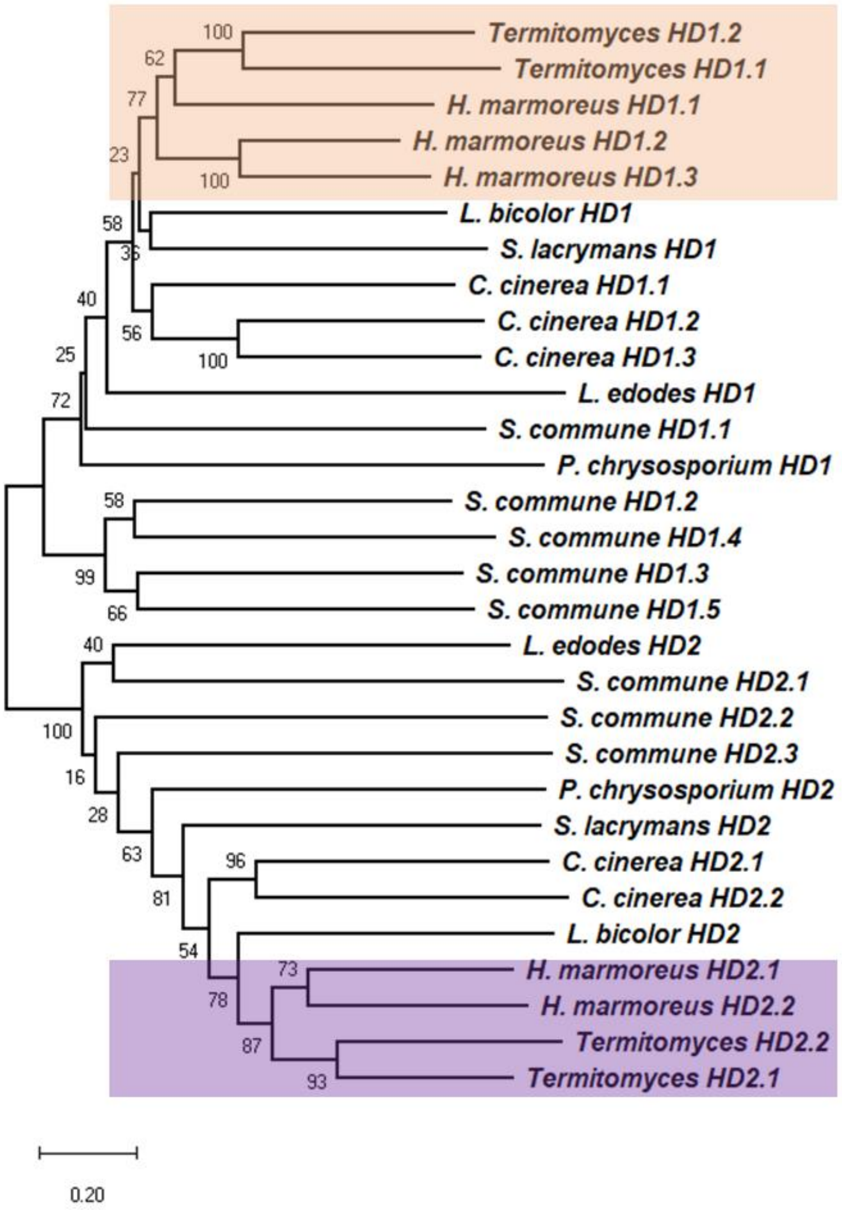

Figure S9. Phylogenetic tree of *HD* among 8 *Agaricales* species.

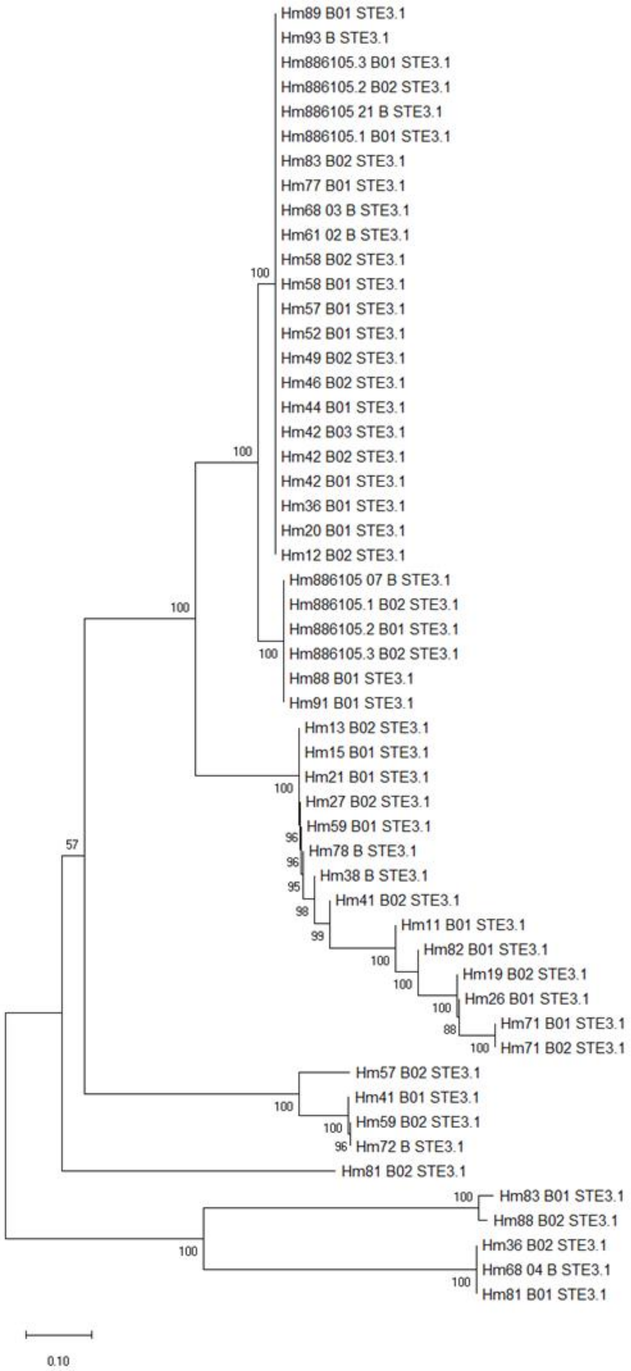

Figure S10. Phylogenetic tree of Pheromone receptor *STE3.1* from all different *H. marmoreus* stains. The B1and B2 represents the allele of the heteronuclear strain, respectively.

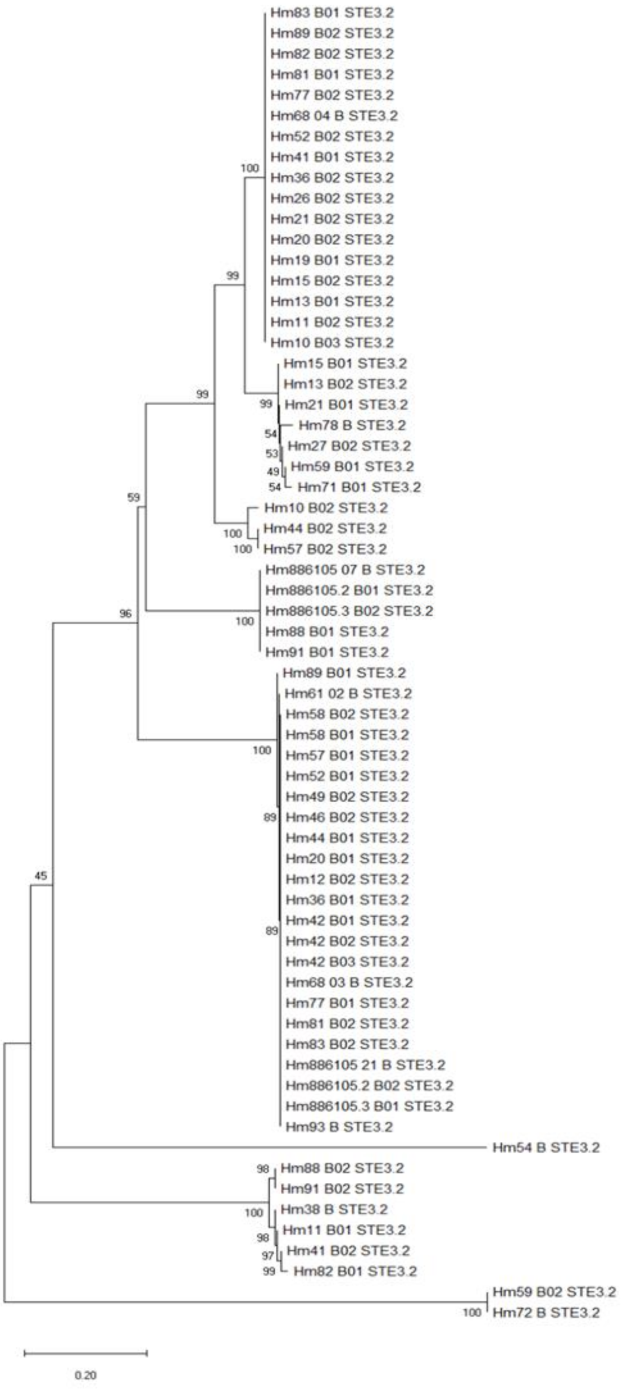

Figure S11. Phylogenetic tree of Pheromone receptor *STE3.2* from all different *H. marmoreus* stains. The B1and B2 represents the allele of the heteronuclear strain, respectively.

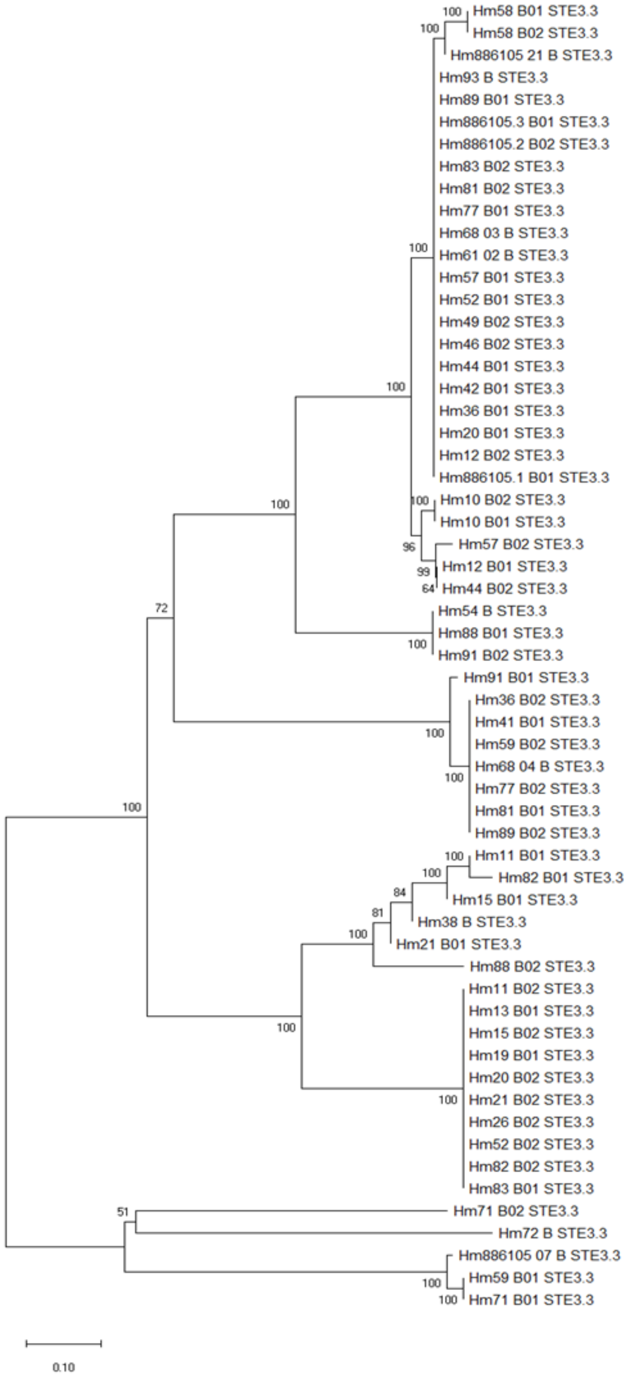

Figure S12. Phylogenetic tree of Pheromone receptor *STE3.3* from all different *H. marmoreus* stains. The B1and B2 represents the allele of the heteronuclear strain, respectively.

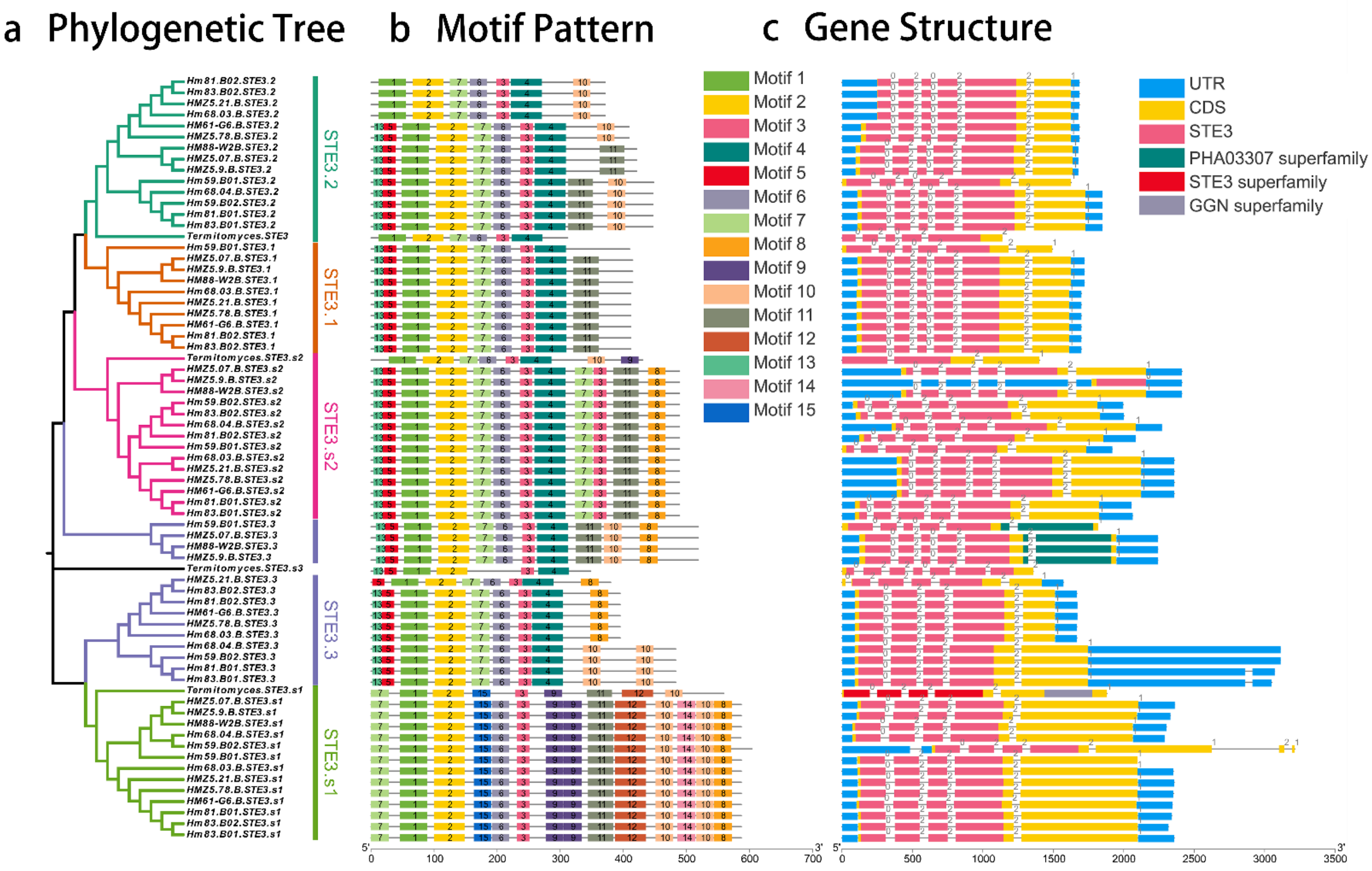

Figure S13. Phylogenetic relationships, gene structure and architecture of conserved protein motifs in *STE3* genes from the different strains. (A). The phylogenetic tree was constructed based on the full-length sequences of *STE3* proteins. Details of clusters are shown in different colors. (B). The motif composition of *STE3* proteins. The motifs, numbers 1–15, are displayed in different colored boxes. (C). Exon-intron structure of *STE3* genes. Blue boxes indicate untranslated 5’- and 3’-regions; yellow boxes indicate exons; black lines indicate introns. The *STE3* domains are highlighted by pink boxes. The number indicates the phases of corresponding introns. The length of protein can be estimated using the scale at the bottom.

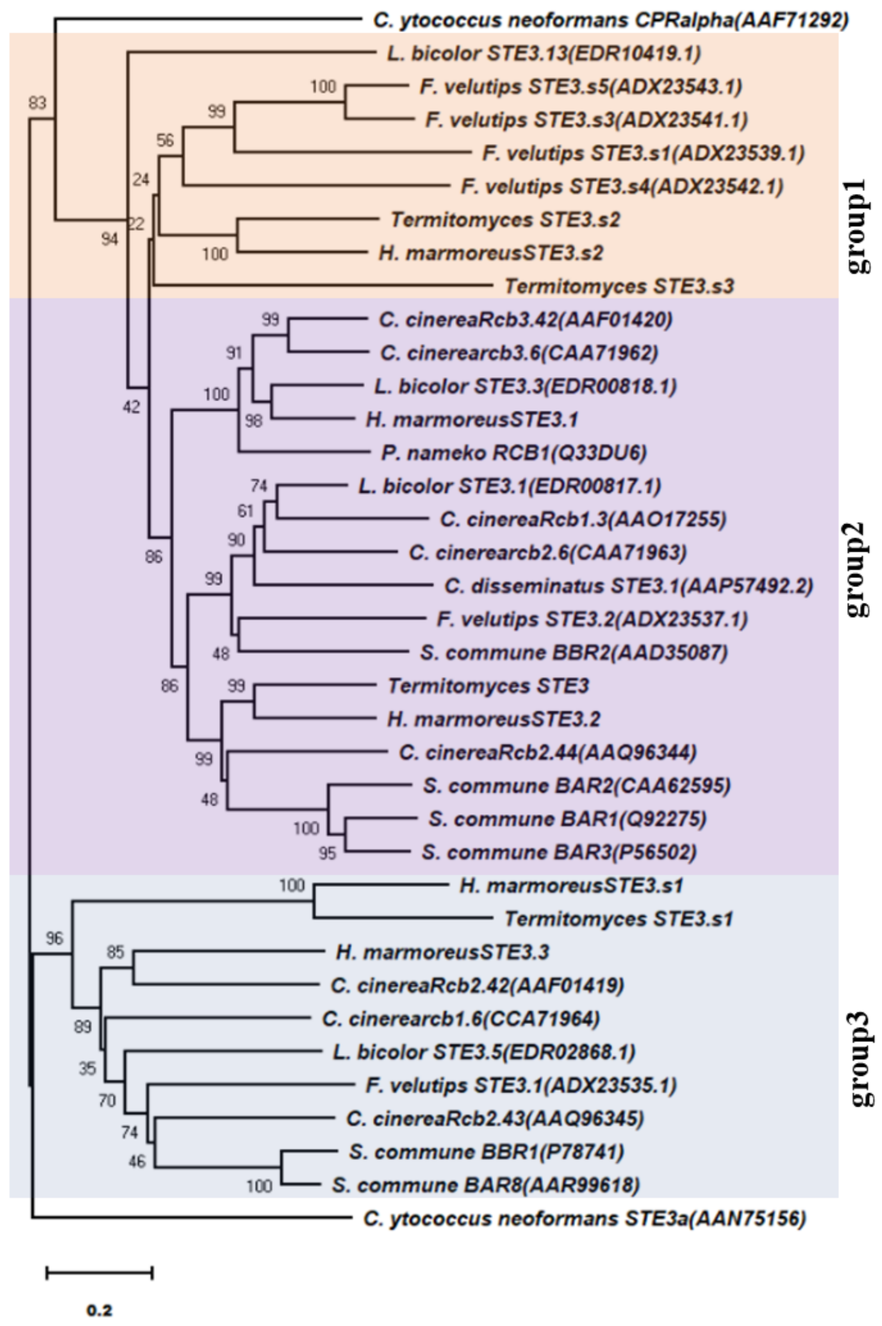

Figure S14. Phylogenetic tree of *STE* genes of *Agaricales* species. The *STE3* genes display the deep-rooted trans-species polymorphism.

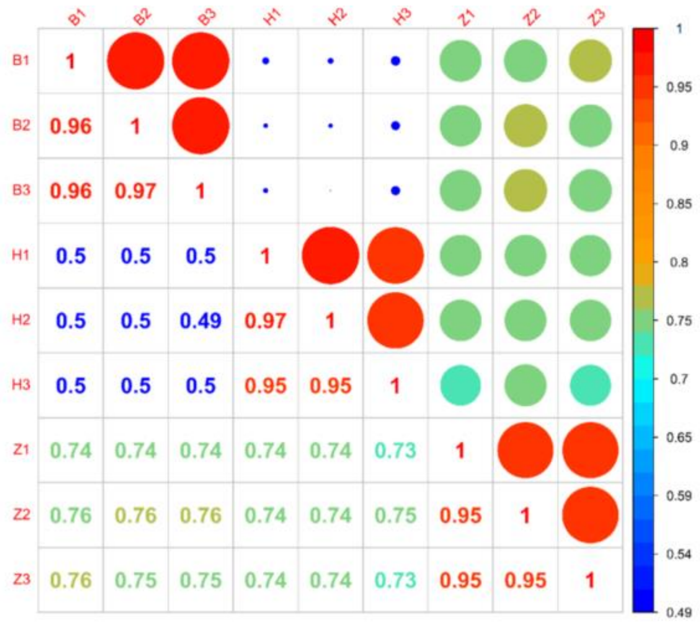

Figure S15. Matrix heat map of Pearson's correlation coefficients for pairs from nine samples of *H. marmoreus*. The color represents the degree of correlation between repetitions in the sample. B indicates the mononuclear strain Hm88-W2, H indicates the mononuclear strain Hm61-G6, Z represents HMZ5, the hybrid strain of Hm88-W2and Hm61-G6.

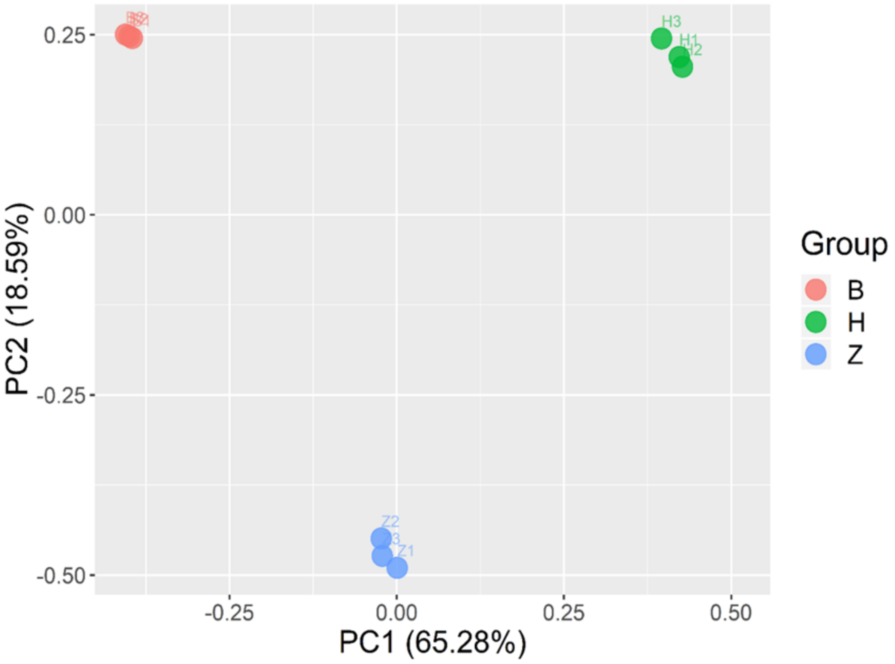

Figure S16. PCA analysis of the gene expression levels for RNA-seq samples from two mononuclear stains and their hybrid stain. B indicates the mononuclear strain Hm88-W2, H indicates the mononuclear strain Hm61-G6, Z represents HMZ5, the hybrid strain of Hm88-W2and Hm61-G6.

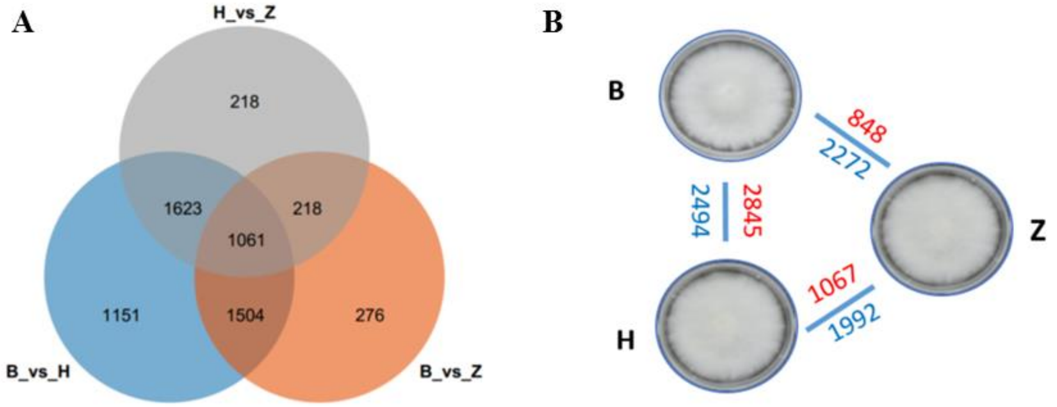

Figure S17. Comparative analysis of differentially expressed genes in mononuclear and binuclear mycelium stages. (A). Venn diagram showing the number of detected proteins overlapping among three samples. (B). Red and blue numbers denote the numbers of significantly upregulated and downregulated gene respectively.

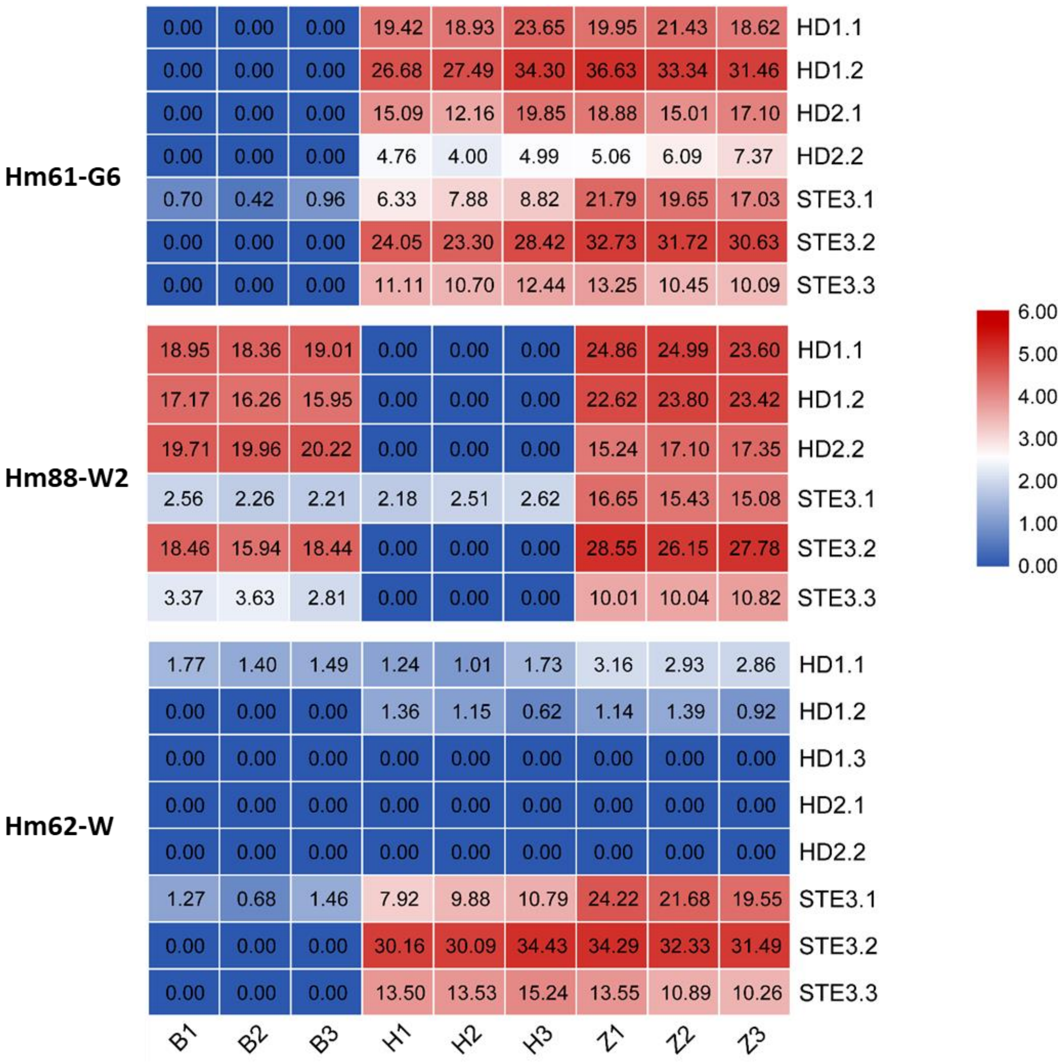

Figure S18. The gene expression were analyzed by reference genomes of different strains. B indicates the mononuclear strain Hm88-W2, H indicates the mononuclear strain Hm61-G6, Z represents HMZ5, the hybrid strain of Hm88-W2and Hm61-G6.

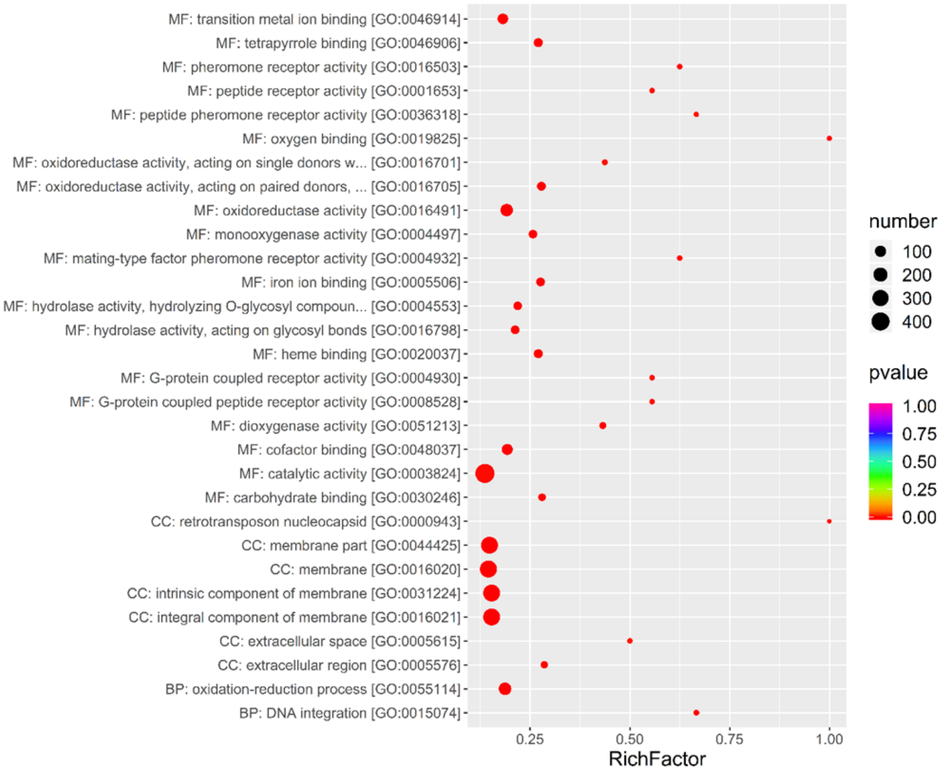

Figure S19. GO enrichment bubble map of down-regulated gene of HMZ5/ Hm61-G6. The vertical coordinate is the enrichment of the top 30 GO functions, and the horizontal coordinate is the Rich factor. P represents significance, and is represented by gradient from low to high (0 to 1). The lower the p value, the higher the GO enrichment degree. The number of genes enriched by GO in DEGs is expressed by bubble size.

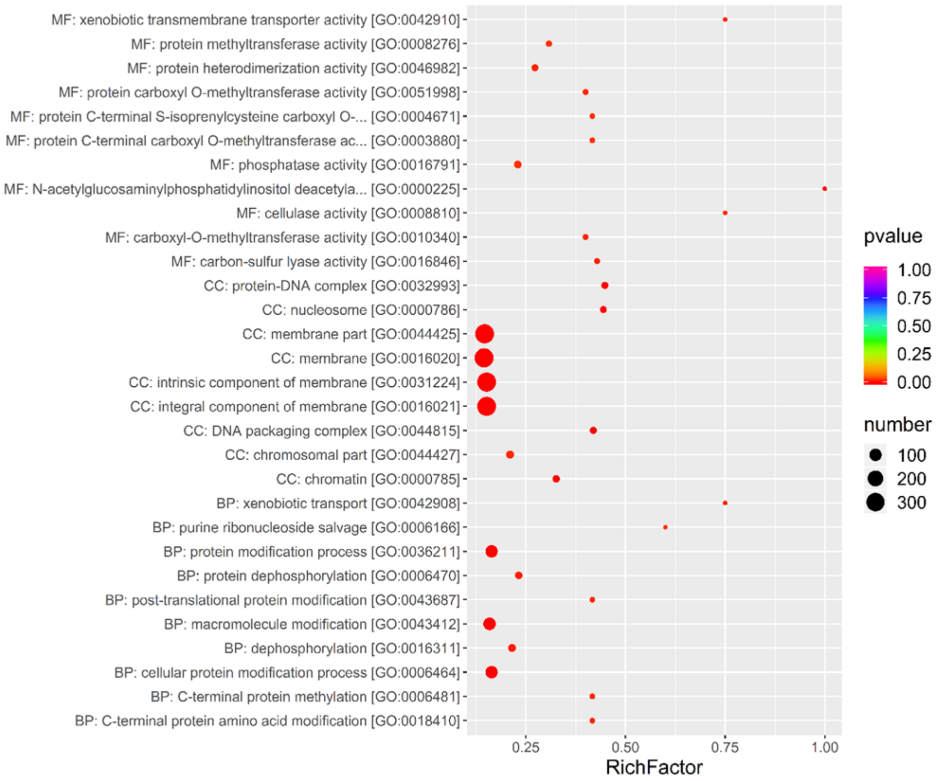

Figure S20. GO enrichment bubble map of down-regulated gene of HMZ5/ Hm88-W2. GO enrichment bubble map of down-regulated gene of HMZ5/ Hm61-G6 stain. The vertical coordinate is the enrichment of the top 30 GO functions, and the horizontal coordinate is the Rich factor. P represents significance, and is represented by gradient from low to high (0 to 1). The lower the p value, the higher the GO enrichment degree. The number of genes enriched by GO in DEGs is expressed by bubble size.

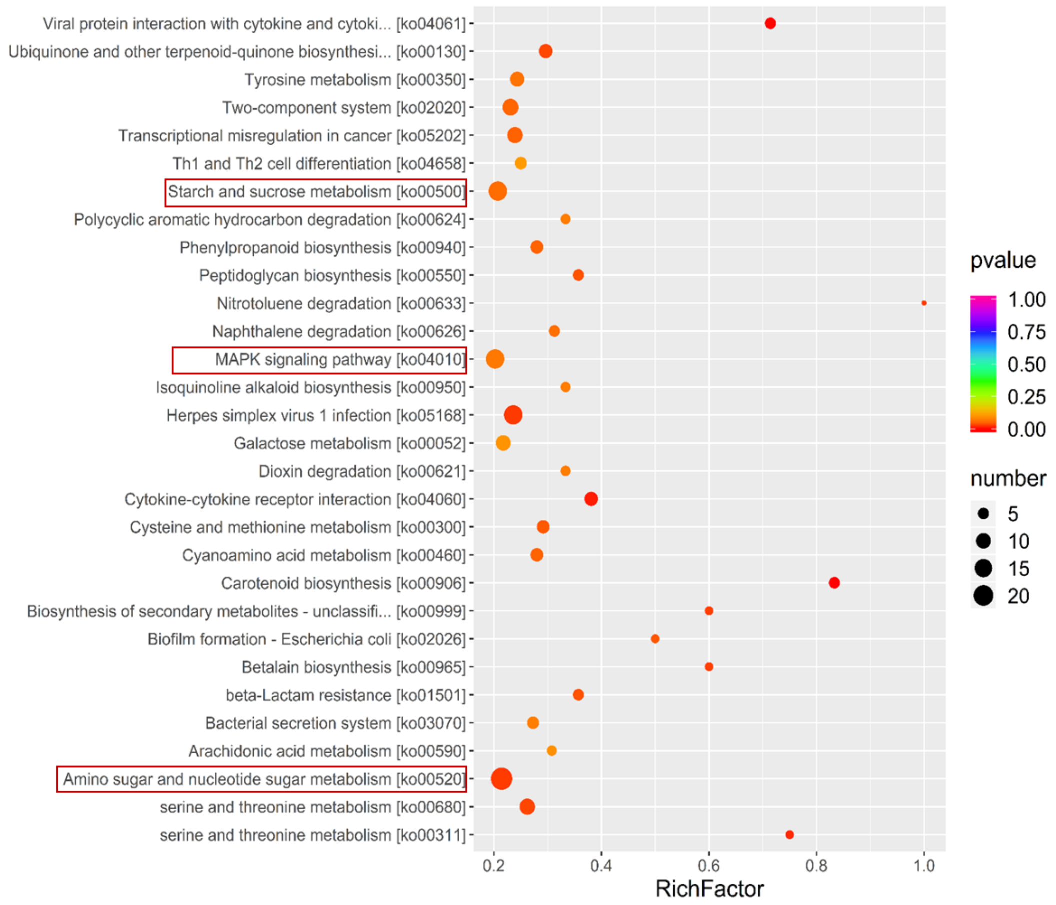

Figure S21. KEGG enrichment bubble map of up-regulated gene of mononuclear / heterocaryon.
